# Five Nuclear Loci Resolve the Polyploid History of Switchgrass (*Panicum virgatum* L.) and Relatives

**DOI:** 10.1371/journal.pone.0038702

**Published:** 2012-06-18

**Authors:** Jimmy K. Triplett, Yunjing Wang, Jinshun Zhong, Elizabeth A. Kellogg

**Affiliations:** Department of Biology, University of Missouri-Saint Louis, Saint Louis, Missouri, United States of America; Field Museum of Natural History, United States of America

## Abstract

Polyploidy poses challenges for phylogenetic reconstruction because of the need to identify and distinguish between homoeologous loci. This can be addressed by use of low copy nuclear markers. *Panicum* s.s. is a genus of about 100 species in the grass tribe Paniceae, subfamily Panicoideae, and is divided into five sections. Many of the species are known to be polyploids. The most well-known of the *Panicum* polyploids are switchgrass (*Panicum virgatum*) and common or Proso millet (*P. miliaceum*). Switchgrass is in section *Virgata*, along with *P. tricholaenoides*, *P. amarum*, and *P. amarulum*, whereas *P. miliaceum* is in sect. *Panicum*. We have generated sequence data from five low copy nuclear loci and two chloroplast loci and have clarified the origin of *P. virgatum*. We find that all members of sects. *Virgata* and *Urvilleana* are the result of diversification after a single allopolyploidy event. The closest diploid relatives of switchgrass are in sect. *Rudgeana*, native to Central and South America. Within sections *Virgata* and *Urvilleana*, *P*. *tricholaenoides* is sister to the remaining species. *Panicum racemosum* and *P. urvilleanum* form a clade, which may be sister to *P*. *chloroleucum*. *Panicum amarum*, *P. amarulum*, and the lowland and upland ecotypes of *P. virgatum* together form a clade, within which relationships are complex. Hexaploid and octoploid plants are likely allopolyploids, with *P. amarum* and *P. amarulum* sharing genomes with *P. virgatum*. Octoploid *P. virgatum* plants are formed via hybridization between disparate tetraploids. We show that polyploidy precedes diversification in a complex set of polyploids; our data thus suggest that polyploidy could provide the raw material for diversification. In addition, we show two rounds of allopolyploidization in the ancestry of switchgrass, and identify additional species that may be part of its broader gene pool. This may be relevant for development of the crop for biofuels.

## Introduction

Reticulate evolution always poses a challenge to phylogenetic reconstruction. Demonstrating reticulate evolution generally requires at least one well-supported nuclear gene tree, and often several. In comparison to the enormous number of molecular phylogenetic studies, relatively few have attempted to disentangle the history of groups in which polyploidy is common, perhaps because of the challenges of working with multiple nuclear markers.

Polyploidy is particularly common and well documented in flowering plants. The history of recent allopolyploids has been amply documented in wild species (e.g. *Spartina* [1], *Tragopogon* [2], *Senecio* [3], inter alia) and under domestication (e.g. bread wheat, durum wheat [4]). In a few cases diversification after polyploidy has been documented although relationships among the polyploids have been unresolved [5]–[8]. Additional rounds of polyploidization leading to higher order polyploids must have occurred, but these have also not been resolved. Older polyploidy has also been discovered based on whole genome duplications at the base of the eudicots, the base of Brassicaceae, and the base of Poaceae, among others [9].

Phylogenetic analysis of single or low-copy nuclear gene sequences is an effective way to study the evolution of allopolyploids. Nuclear gene trees used along with chloroplast gene trees have been used to confirm the existence of allopolyploids, to identify genome donors, and to examine gene evolution within polyploids. Several recent studies have used this method successfully to infer complex reticulate relationships among plant species, e.g., in *Paeonia* [10], *Gossypium* [8], *Glycine* [11], *Elymus* [6], [7], and *Hordeum* [12].

Here we present a detailed analysis of a large clade within the genus *Panicum*, demonstrating that the use of several nuclear markers coupled with chloroplast markers can disentangle a pattern of divergent evolution followed by reticulation, followed in turn by divergence and a subsequent round of reticulation. It is likely that many angiosperms have similarly intricate histories, but to our knowledge this phylogenetic reconstruction may be the most complex yet presented with this level of resolution and clade support.

**Table 1:** Species of *Panicum* included in this study.

Section	Species	Distribution	2 n	References for chromosome numbers
***Dichotomiflora***				
	*P. aquaticum*	North America, Central America, South America, Caribbean.	72	[85]
	*P. aff. aquaticum*	Costa Rica	–	
	*P. dichotomiflorum*	Southwestern and southeastern Europe, temperateAsia, New Zealand and north-central Pacific, North America, Central America, South America, Caribbean.	54 (36)	[86]–[90]
	*P. elephantipes*	Mexico, Central America, South America, Caribbean.	30	[91]
	*P. gouinii*	Southeast USA, Mexico, Caribbean, Brazil, southern South America.	20, 36	[92], [93]
	*P. pedersenii*	Southern South America.	–	
***Rudgeana***				
	*P. campestre*	Western South America, Brazil.	–	
	*P. cayennense*	Mexico, Central America, Caribbean, northern and western South America, Brazil.	–	
	*P. cervicatum*	South America.	–	
	*P. rudgei*	Mexico, Central America, Caribbean, northern and western South America, Brazil.	18	[85]
***Virgata***				
	*P. amarulum*	Eastern and southern USA, Mexico, Central America, Caribbean.	36	[54]
	*P. amarum*	Eastern and southern USA, Mexico, Central America, Caribbean.	54	[54]
	*P. tricholaenoides*	South America.	36	[92], [93]
	*P. virgatum*	Soviet Middle Asia (introduced), north-central Pacific, Canada, USA, Mexico, Caribbean, southern South America.	36, 54(18–108)	[32], [49], [88], [90], [94]
	*P. virgatum subsp. cubense*	USA, Caribbean	–	
***Urvilleana***				
	*P. chloroleucum*	Southern South America.	–	
	*P. racemosum*	Australia (introduced). Brazil and southern South America.	36	[92], [93]
	*P. urvilleanum*	Southwest USA, Mexico, southern South America.	36	[92], [93]
***Panicum***				
	*P. bergii*	South America.	–	
	*P. capillare*	Widespread and weedy. Europe, Macaronesia, temperate Asia, India, Australia, New Zealand, southwestern Pacific, Canada, USA, Mexico, Caribbean, southern South America.	18	[88], [95]–[99]
	*P. miliaceum*	Widespread and cultivated. Europe, Africa,Macaronesia, temperate and tropical Asia, Australia,New Zealand, Pacific, North America, South America,Caribbean.	36	[86], [86], [100]–[106]
	*P. nephelophilum*	North-central Pacific.	–	
	*P. stramineum*	Southwest USA, Mexico, western South America,Brazil.	36	[107]
***Incertae sedis***				
	*P. mystasipum*	Brazil.	–	
	*P. olyroides*	South America.	36	[82]

Chromosome numbers, if indicated, are based on a review of the literature.

*Panicum sensu stricto* is a genus of grasses (Poaceae) with about 100 species distributed throughout the tropical and temperate regions of the Old and New Worlds; many species are polyploid. *Panicum sensu lato* is clearly polyphyletic and is now almost completely dismembered [13]–[20] but *Panicum s.s.* is amply supported as monophyletic by data from multiple chloroplast genes [13], [21], and from the nuclear gene phytochrome B [22]; all species have a basic chromosome number of *x* = 9. In addition, *Panicum s.s.* is morphologically coherent. Shared derived characters include the presence of simple or compound papillae toward the apex of the palea (floral bract), and the C_4_ photosynthetic pathway with an NAD-malic enzyme as the primary decarboxylating enzyme (i.e. C_4_ NAD-ME subtype). This subtype is connected with distinctive leaf anatomy, so all members of the genus are identifiable by leaf cross section alone [23], [18].

*Panicum s.s.* has been divided into five sections on the basis of morphology, and to a limited extent supported by molecular data [13]. Sections *Panicum*, *Dichotomiflora*, and *Virgata* are distributed worldwide, with species in America, Africa, Europe, Asia, and Oceania, while *Rudgeana* occurs from South to Central America, and *Urvilleana* is restricted to North America (with one possibly related species present in northern Africa) [24], [13]. Sections *Virgata* and *Urvilleana* together form a well-supported group in the previous *ndhF* molecular phylogeny [13]. Additionally, several species have distinctive characteristics and remain unplaced as to section, including *P. mystasipum* and *P. olyroides*.

Switchgrass (*P. virgatum*, section *Virgata*) is an economically and ecologically important species of *Panicum*. It is a C_4_ perennial, native to North American tall grass prairies [25], that has long been used for conservation plantings and as a forage crop, and is currently being developed as a biomass energy crop in the United States for use on marginal cropland [26]–[29]. Switchgrass is self-incompatible and outcrossing, and is highly heterozygous [30], [31]. Most members of the species are either tetraploid or octoploid; the one report of a diploid plant [32] has not been confirmed [33]. Normal diploid chromosome pairing occurs at meiosis for all tetraploid and octoploid plants that have been examined [34] and inheritance in the tetraploids is disomic [35] suggesting an allopolyploid origin of the species. *Panicum virgatum* var. *cubense* and *P. virgatum* var. *spissum* are names applied to plants apparently at the end points of geographic clines, while plants from eastern New Mexico, western Texas, and northern Mexico that exhibit larger spikelets are sometimes recognized as *P. havardii* [36].

**Table 2:** Flow cytometry results.

Taxon	Accession	Ploidy estimate
***P. amarulum***	aml419 (2)	4x
	aml421901	4x
	aml476814−1	4x
	aml476815−1	4x
	aml476815−2	4x
***P. amarum***	ama7 (2)	8x
	ama8	6x
	ama9	6x
	ama10	6x
	ama11	6x
	ama12	6x
	ama561721	6x
	ama645599	6x
***P. virgatum (lowland ecotype)***	vir441	4x
	vir315723	4x
	vir414065	4x
	vir414070	4x
	vir421521	4x
	vir421999	4x
	vir422006	4x
	vir422016	4x
	vir476291 (2)	4x
	vir607837	4x
*P. virgatum subs. cubense*	cub315728 (2)	4x
***P. virgatum (upland ecotype)***	vir16409	8x
	vir315724	8x
	vir315725	4x
	vir337553	8x
	vir414066	8x
	vir414067	8x
	vir414069	8x
	vir421138	8x
	vir421520	8x
	vir431575	8x
	vir469228	8x
	vir476292	8x
	vir476293	4x
	vir476296	4x
	vir476297	8x
	vir549094	8x
***P. aff. aquaticum***	unkCR3	6x

Predicted ploidy levels as measured by flow cytometry. Estimates based on multiple samples of the same individual are indicated with parenthetical numbers; those based on different individuals representing the same accession are indicated with hyphenated numbers.

Switchgrass includes two major ecotypes, upland and lowland, that are distinguished ecologically and morphologically [37]–[41], and also tend to form clades in molecular studies [42]–[47]. The lowland ecotype is uniformly tetraploid, whereas plants of the upland type may be tetraploid or octoploid; the latter two ploidy levels commonly co-occur [38], [40], [41], [43], [48]–[52]. The tetraploids are interfertile and chromosomes of the hybrids pair normally [34], [37]. Hexaploid plants of *P. virgatum*, in contrast, are rare [37], [53]. The nuclear genome of a lowland genotype of *P. virgatum*, Alamo AP13, is currently being subjected to full genome sequence analysis (J. Bennetzen, pers. comm.), and a full genetic map is available [35].

Closely related to *P. virgatum*, and sometimes intergrading with it, are *P. amarum* and *P. amarulum*. The latter two are often considered conspecific (as *P. amarum* variety or subspecies *amarum* and *P. amarum* var. or subsp. *amarulum*) [36]. *Panicum amarulum* is tetraploid, while *P. amarum* includes hexaploids and occasionally octoploids. The two taxa co-occur in coastal regions of the Eastern U.S., and have been studied in most detail in Delaware [53] and North Carolina (Youngstrom and Kellogg, unpublished). Along the east coast, plants of *P. amarum* grow on the foredunes, within easy reach of salt spray. They are robust plants, with dense contracted inflorescences, powerful rhizomes, and waxy leaves that give them a handsome bluish color. They bloom later than *P. virgatum*, and are partially sterile. In contrast, *P. virgatum* grows back from the beach, often in or near sparse woods. The plants are caespitose rather than strongly rhizomatous, the inflorescence is relatively sparse with spreading branches, and the leaves are less waxy. *Panicum amarulum* is less distinct morphologically and its relationship to *P. virgatum* and *P. amarum* is unclear. Palmer [54] has suggested that hybridization may contribute to the morphological variation.

The current study aims to unravel the history and formation of polyploids among switchgrass and its relatives in sections *Virgata* and *Urvilleana*, with additional species of *Panicum s.s*. included for comparison. Specifically, we asked the following questions: (1) What are the phylogenetic relationships among species in *Panicum s.s.*, and in particular, with respect to polyploidy?; (2) Did speciation occur before or after the origin of polyploid species?; and (3) Which lineages contributed to current day polyploids in switchgrass? We have discovered that sections *Virgata* and *Urvilleana* together are the result of a single ancestral polyploid event, which was followed by divergent speciation at the tetraploid level. We thus document divergent evolution, followed by reticulation, followed again by divergence, followed by another round of reticulation.

**Table 3:** DNA primers and PCR parameters used for amplification and sequencing.

Region	Location	Primer sequences (5′ to 3′)	PCR Parameters	Reference
***rps16–trnQ***	Plastid	1F: GCA CGT TGC TTT CTA CCA CA	95°C, 2 min; 35x (95°C, 1 min; 50°C, 10 sec; +15°C, 0.3°C/s; 65°C, 5 min); 65°C, 5 min.	[60, this paper (internal primers)]
		1574R: ATC CTT CCG TCC CAG ATT TT		
		*Internal primer:* 16Q308f:CGA CTC TTC CCC AAC AAA TAA AC		
		*Internal primer:* 16Q752r: GCA AAA ACG ATC TCGATC TGT G		
***trnC-rpoB***	Plastid	trnC: TGG GGA TAA AGG ATT TGC AG	94°C, 2 min; 35x (96°C, 1 min; touchdown 56–46°C, 2 min; 72°C, 3 min); 72°C, 5 min.	[60, 108, this paper (internal primers)]
		rpoB: ATT GTG GAC ATT CCC TCR TT		
		*Internal primer:* jt382for:GCA TTA TTA TCT ATG GAT CCC CC		
		*Internal primer:* jt744rev:TAG TTC GAT TTA GAA TAG CAC GCT TA		
***Adh1***	Chromosome 11	Adh1-F5: TCC CGT GTT CCC TCG GAT CTT C	95oC, 2 min; 35x (95oC, 1 min; 57oC, 1 min; 72oC, 1 min); 72oC, 5 min	[61]
		Adh1-R3: GTC ACA CCC TCT CCA ACA CTC T		
***Knotted1***	Chromosome 3	Kn1-F: CCG CAC TAC TAC TCG CTC CT	95°C, 2 min; 35x (95°C, 45 s; 55°C, 45 sec; 72°C, 1.5m); 72°C, 5 min	This paper.
		Kn1-R: GCC AGA GGA AAG GAT ATT GC		
***Pabp1***	Chromosome 4	Pv17357 for: GCT TGT CCA TAG AAG AGT TG	95°C, 2 min; 35x (95°C, 1 min; 53°C, 1 min; 72°C, 1.5 min); 72°C, 5 min	This paper.
		Pv17357 rev: GCC ACT GTA TGT TGC ATT TG		
		P4F1+P4AR – *A genome screen:*		
		PABP4F1:ATAGGAGGGTACATTGGAAG		
		PABP4AR:GTTTACTATAGATTGTTACAAGTG		
		P4BF+P4R1− *B genome screen:*		
		PABP4BF: ATGCCTCTTYAGACCAAAC		
		PABP4R1: CAATGTTACAGGTATATTCCTC		
***PvCel1***	Chromosome 9	b14447_2_for: CWG AAG CYG TCA TTT GTG G	95°C, 2 min; 35x (95°C, 1 min; 54.5°C, 45 sec; 72°C, 1 min); 72°C, 5 min	This paper.
		b14447_2_rev: CGC CCC TCT GTG GTG TAC		
***PvCel2***	Chromosome 3	g12492_3_for: GAT GTA CTT YGC CAC RGG GAA	95°C, 2 min; 35x (95°C, 1 min; 54.5°C, 45 sec; 72°C, 1 min); 72°C, 5 min	This paper.
		g12492_3_rev1: TGC GGA GGA CTT CTR TRG TGT		

Asterisks indicate published primer sequences that were modified for this study; underlines indicate modified nucleotide sites. Chromosomal locations of nuclear genes are based on rice.

## Materials and Methods

### Plant Material and DNA Extraction

Taxa included in this study are summarized in Table 1, and individual accessions are listed in Appendix S1. The sample encompasses the morphological diversity and geographical distribution of *Panicum*, but focuses particularly on sections *Virgata* and *Urvilleana*. Therefore we included multiple individuals of species in the latter two sections. None of the species investigated here is endangered or protected. Some specimens were collected in the field in the U.S., Mexico, Costa Rica, and South America. For the U.S. specimens, no specific permits were required; the land on which the plants were growing was neither privately owned nor protected. All necessary country-specific permits were obtained for the non-U.S. material. Numerous accessions of *P. amarulum*, *P. amarum,* and *P. virgatum* were obtained from the Germplasm Resources Information Network (GRIN; http://www.ars-grin.gov/), representing different ecotypes and ploidy levels from throughout the native range of switchgrass. Most of this material originated from natural populations [48], and was collected under the auspices of the USDA. Plants were grown to flowering, identification was verified, and voucher material deposited at the Missouri Botanical Garden. Leaf samples from natural populations were dried in silica gel. *Pennisetum alopecuroides*, *Setaria palmifolia*, *Setaria viridis*, and *Urochloa plantaginea* were chosen as outgroup taxa based on previous phylogenetic studies [13].

DNA was extracted by one of the following methods: an SDS protocol using a lysis buffer of 100 mM Tris, 500 mM sodium chloride, and 50 mM EDTA +20% SDS, with a protein precipitation using 5 M potassium acetate (modified from Junghans and Metzlaff [55]); a large scale CTAB protocol [56] using CsCl2 gradients [57]; a small scale CTAB protocol [58], or Qiagen DNeasy Plant-mini DNA extraction kits (QIAGEN, Valencia, CA., USA).

**Table 4:** Statistics and evolutionary models for separate data partitions.

Partition	N	Total char.	PIC	MPTrees	MPLength	CI	RI	Model
*rps16-trnQ*	82	1739	55	378	156	0.7901	0.9524	GTR+I+G
*trnC-rpoB*	82	1465	55	198	141	0.8649	0.9708	GTR+I+G
*cpDNA (plus indels)*	82	3222	128	66	323	0.8066	0.9569	n/a
*Adh1*	191	1279	245	2061100	713	0.6154	0.9452	TrN+G
*Knotted1*	215	986	191	2000000*	463	0.6757	0.9701	SYM+G
*Pabp1*	189	1128	371	470386	797	0.7299	0.9676	TVM+I
*PvCel1*	89	757	109	1008	272	0.7722	0.9578	HKY+G
*PvCel2*	84	751	155	4523800	398	0.6784	0.9201	HKY+G
*nDNA (combined)*	83	4388	1009	2000000*	2050	0.7416	0.9364	n/a
*A+B combined* *(knotted1+ pabp1)*	30	3299	194	12	333	0.8918	0.9659	n/a

PIC  =  parsimony informative characters. PIC parenthetical indicates the number of parsimony informative characters within the temperate clade. MP  =  maximum parsimony; CI  =  consistency index, excluding uninformative characters; RI  =  retention index. Models are based on the Hierarchical Likelihood Ratio Test implemented in jModelTest. MP tree numbers with asterisks indicate the maximum number that was saved.

### Flow Cytometric Determination of Ploidy

Chromosome numbers have been published for many species included in this study (Table 1), but ploidy levels for switchgrass and its close relatives *P. amarulum* and *P. amarum* are variable. We used flow cytometry to determine the cytotype for most accessions of *P. virgatum* and *P. amarum* plus four individuals of *P. amarulum* and one individual of *P.* aff. *aquaticum*. Leaf tissue was collected from greenhouse plants and mailed to the Iowa State University Flow Cytometry Facility, where samples were processed and analyzed on a Beckman-Coulter Epics XL-MCL flow cytometer (Fullerton, California, USA). Cytotypes were determined by measuring the propidium iodide fluorescence (488 nm excitation) of ∼3000 nuclei, using chicken eyrthrocyte nuclei (CEN) as a genome size reference. Fluorescence values were converted to chromosome number using tetraploid *P. virgatum* as a standard (Table 2).

### Locus Amplification, Molecular Cloning, and Sequencing

#### Plastid loci

We selected the plastid loci *rps16-trnQ* and *trnC-rpoB*, which are known to contain relatively high sequence diversity [59], [60]. Both are noncoding spacer regions. Primers and PCR protocols are listed in Table 3. PCR products were purified using 5 U Antarctic phosphatase (New England Biolabs, Beverly, Massachusetts, USA) and 10 U exonuclease I (New England Biolabs) at 37°C for 30 min, followed by inactivation of the enzymes at 80°C for 15 min. Both strands of the amplified products were sequenced using the ABI-Prism Big Dye Terminator sequencing method (Version 3.1; Applied Biosystems). Sequence reactions were run on an Applied Biosystems ABI Hitachi 3730XL DNA Analyzer at the Huck Institutes of the Life Sciences (Penn State, http://www.huck.psu.edu/facilities).

**Figure 1:**
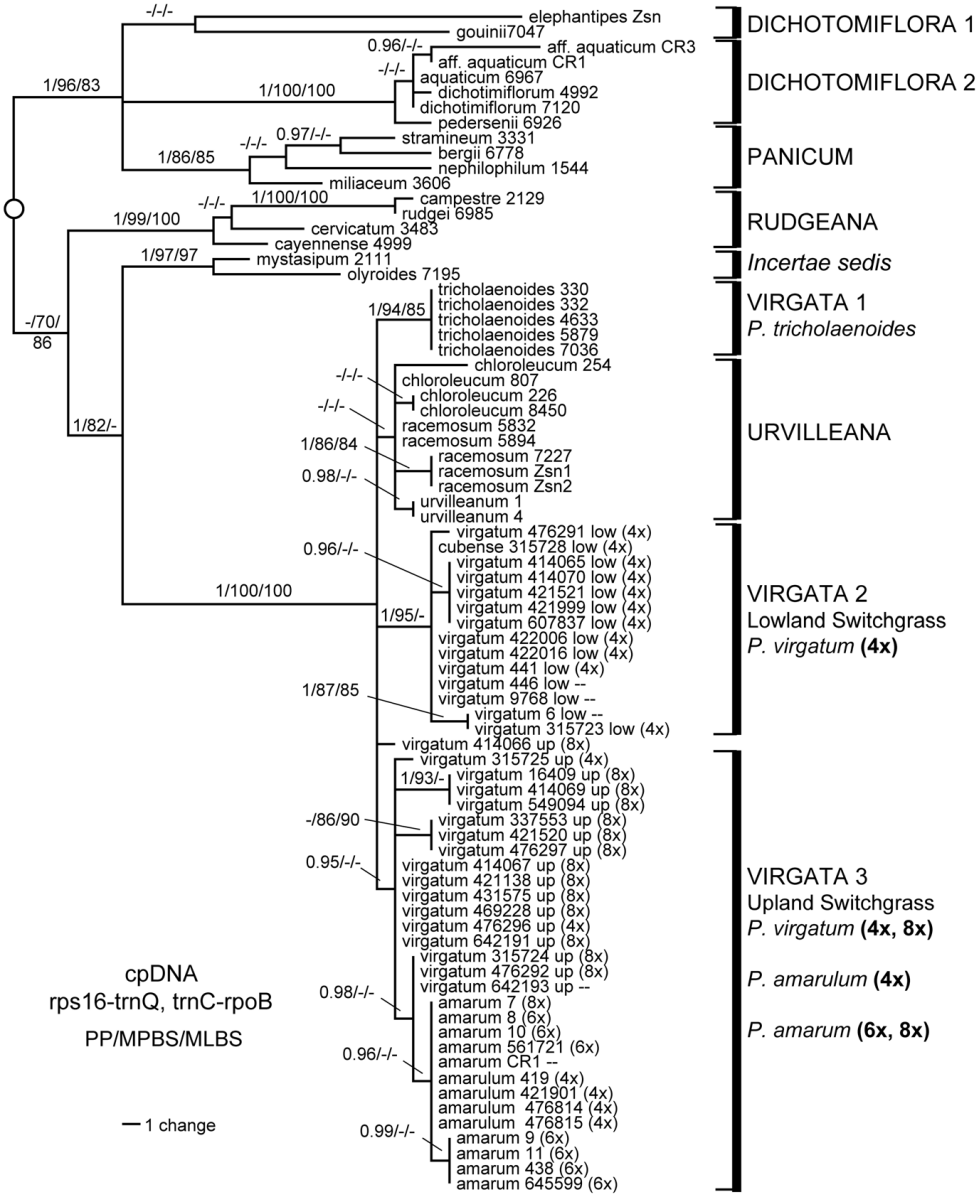
Bayesian phylogram based on combined cpDNA data. Support values are Bayesian posterior probability/maximum parsimony bootstrap/maximum likelihood bootstrap. Names in all caps to the right of brackets indicate sections of *Panicum* s.s. Outgroups have been omitted for clarity.

#### Nuclear loci

We selected nuclear regions that are predominantly single copy in grasses, making it possible to establish orthology and track homoeologues arising through allopolyploidy. After a preliminary survey of eight regions, we selected *alcohol dehydrogenase*1 (*adh*1), *knotted*1, *poly-A binding protein1* (*pabp*1), *cellulase1* (*PvCel*1), and *cellulase2* (*PvCel*2) based on their level of variation and ease of amplification. Regions Os1283 [61], OsC1_15 [62], a Drm3-like protein, and numerous cellulase regions were tested and excluded because they lacked sufficient informative characters (Triplett, unpublished data). Based on genomic studies of grass crop species, each marker appears to be on a different chromosome (Table 3), except that *PvCel*2 and *knotted*1 are both on chromosome 3 of rice. We assume that each of these five regions provides an independent estimate of phylogeny.

**Figure 2:**
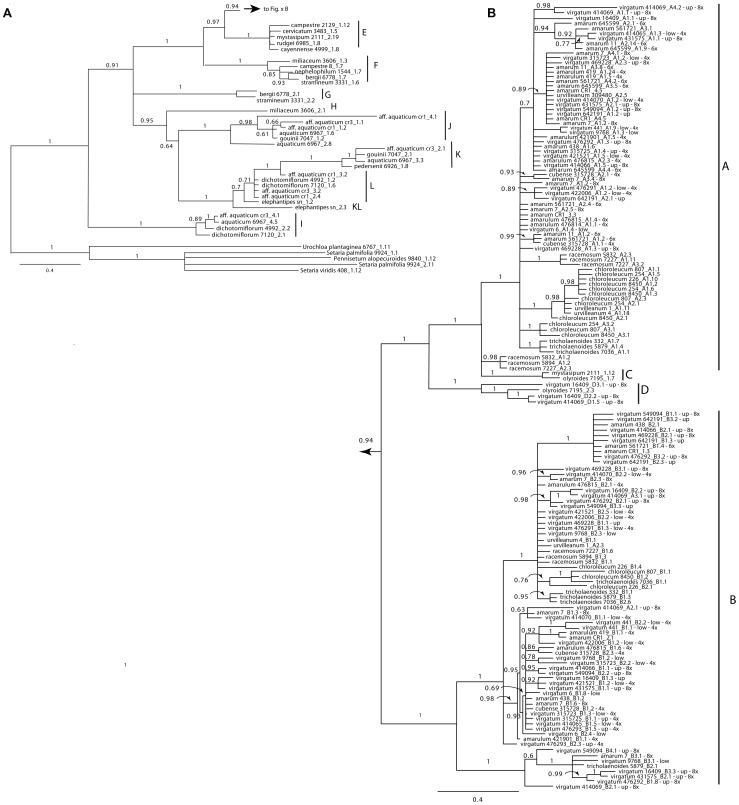
Bayesian phylogram for the unabridged *adh1* dataset, including outgroups and placement of all sequences obtained from *Panicum*; numbers above branches indicate posterior probabilities above 0.5. Letters indicate well-supported clades, inferred to correspond to genomic groups. Taxon labels are in the format: virgatum 414069_A4.2 - up −8x where virgatum 414069 is *P. virgatum* (PI 414069), A4.2 indicates sequence type A4, for which we recovered 2 clones, “up” is the upland ecotype, and 8x is the inferred ploidy level inferred from flow cytometry.

**Figure 3:**
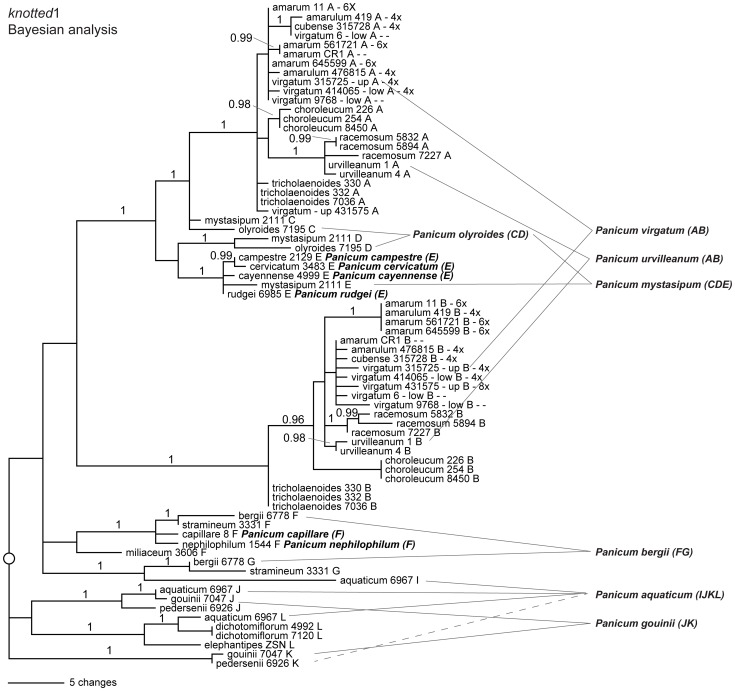
Bayesian phylogram for the *knotted1* abridged dataset. Selected examples of polyploid individuals are indicated. Outgroups have been omitted for clarity.

Three of the loci have been used previously in phylogenetic studies of grasses. Intron 3 of *adh*1 was used in studies of rice [61]. The first two introns and the second exon of *knotted*1, including about 650 bp, was used in an investigation of the “bristle clade” of grasses [63]. *Pabp*1 has been used as a phylogenetic marker in rice [60] and bamboo (Triplett, unpublished data).

The cellulase markers were developed specifically for this study. Endo-1,4-β-glucanases (cellulases) (also termed EGases, EC 3.2.1.4) are well-characterized in bacteria, fungi, plants, and animals, and are of interest for cellulose degradation [64]–[66]. The gene products include an N-terminal extension, a glycosyl hydrolase core, and a membrane-spanning domain [67], [68]. The cellulase genes have multiple introns and potentially provide ample variation for phylogeny reconstruction. We selected Introns 2 and 3 of *PvCel*1, corresponding to rice gene Os09g0533900, and introns 3 and 4 of *PvCel*2, corresponding to rice gene Os03g0736300, because they amplified easily and produced relatively high numbers of informative characters in preliminary analyses.

**Table 5:** Divergent genomes within Panicum s.s. as inferred from nDNA clades.

Genome	Section	Species**
A	*Urvilleana, Virgata*	*P. amarum, P. amarulum, P. chloroleucum, P. racemosum, P. tricholaenoides, P. urvilleanum, P. virgatum*
B	*Urvilleana, Virgata*	*P. amarum, P. amarulum, P. chloroleucum, P. racemosum, P. tricholaenoides, P. urvilleanum, P. virgatum*
C	Incertae sedis	*P. mystasipum, P. olyroides*
D	Incertae sedis	*P. mystasipum, P. olyroides*
E	*Rudgeana,* Incertae sedis	*P. cayennense, P. cervicatum, P. campestre, P. rudgei, P. mystasipum*
F	*Panicum*	*P. bergii, P. capillare, P. miliaceum, P. nephelophilum, P. stramineum*
G*	*Panicum*	*P. bergii, P. stramineum*
H*	*Panicum*	*P. miliaceum*
I*	*Dichotomiflora*	*P. aquaticum, P. dichotomiflora, P. pedersenii*
J*	*Dichotomiflora*	*P. aquaticum, P. gouinii, P. pedersenii*
K*	*Dichotomiflora*	*P. aquaticum, P. elephantipes, P. gouinii, P. pedersenii*
L*	*Dichotomiflora*	*P. aquaticum, P. dichotomiflora, P. elephantipes, P. pedersenii*
kl*	*Dichotomiflora*	*P. elephantipes*

* Asterisks indicate that the genome was found in some but not all species in a particular taxonomic section. (In contrast, genomes A and B were found in all sampled members of sections *Urvilleana* and *Virgata*, while genome F was found in all sampled members of section *Panicum*).

** Diploid species are underlined.

Using the software Primaclade [69], we designed new primers for *knotted*1 and the cellulases based on alignments of cDNA sequences for maize and rice and EST data for *Panicum virgatum*. Primers were anchored in exons and designed to produce amplicons of ∼800–1100 bp. The primers used for PCR and sequencing reactions are listed in Table 3. For *pabp*1, we also designed homoeologue-specific primers.

**Figure 4:**
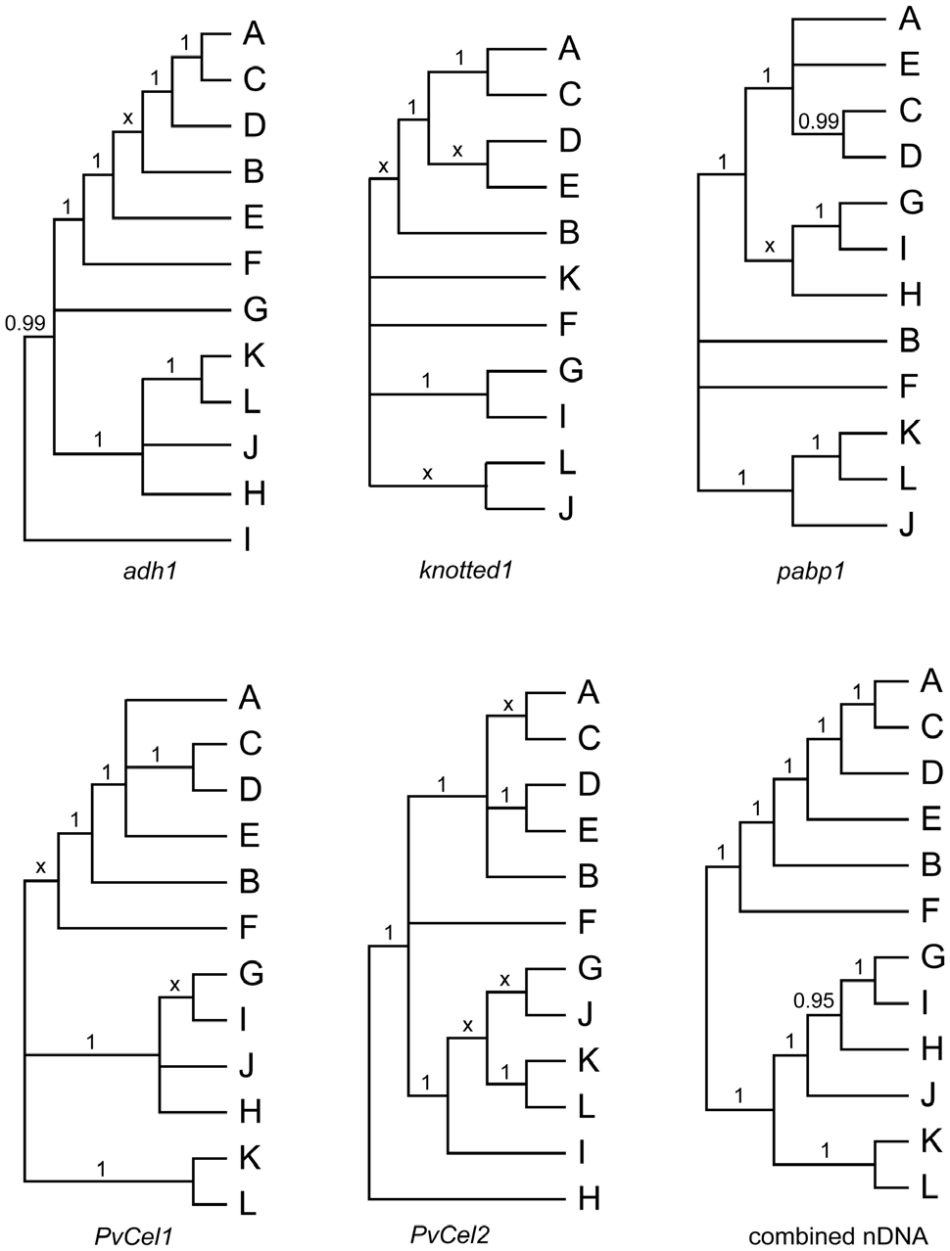
Summary trees for each of the nDNA regions and the combined nDNA tree, based on data sets with ∼83 accessions. We use letters to indicate well-supported clades, and infer that these correspond to genomic groups.

PCR amplification used the following protocol: initial denaturation phase of 95°C for 2 min, 35 cycles of amplification at 95°C for 1 min, primer-specific annealing temperature (Table 3) for 1 min, 72°C elongation for 1 min, followed by a final elongation phase of 72°C for 5 min. PCR reactions were conducted in a 25 µL volume of 1 × *Taq* polymerase buffer, 100–500 ng total genomic DNA, 2.0 mM MgCl 2, 0.4 μM of both forward and reverse primers, 1.00 mM dNTPs (0.25 mM each dNTP), and 2 units of *Taq* polymerase (Bioline USA, Randolph, Massachusetts, USA). In a few cases, PCR reactions were conducted using reagents from the GoTaq Green Master Mix kit (Promega, Madison, Wisconsin, USA).

PCR products were purified using a Qiagen gel extraction kit (QIAGEN, Valencia, CA, USA), cloned into pGEM-T vectors (Promega, Madison, Wisconsin, USA), and transformed into JM109 competent cells (Promega, Madison, Wisconsin, USA) following the manufacturer’s protocol, except that all reaction volumes were halved. Transformed cells were plated and selected via a blue-white screen on LB agar (Sigma) with X-Gal (Promega), isopropyl-beta-thio-galactoside (IPTG; Promega), and ampicillin (Sigma). To assess PCR errors and allelic sequences, 8–24 colonies were selected from each individual. These transformed colonies were grown for 16 h in LB broth. Plasmids were isolated using 5Prime Manual FastPlasmid Mini Kit (Fisher Scientific Company, LLC) and inserts were sequenced with vector primers T7 and SP6 following the ABI-Prism Big Dye Terminator sequencing method (version 3.1; Applied Biosystems, Foster City, California, USA). Sequence reactions were run on an Applied Biosystems ABI Hitachi 3730XL DNA Analyzer at the Huck Institutes of the Life Sciences (Penn State, http://www.huck.psu.edu/facilities).

**Table 6:** Summary of inferred ploidy levels and genomic compositions for taxa in the current study.

Section (clades)	Ploidy	Genomic components
***Virgata*** ** (A, B)**		
*P. amarum*	6x, 8x	AB
*P. amarulum*	4x	AB
*P. virgatum*	4x, 8x	AB
*P. virgatum subsp. cubense*	4x	AB
*P. tricholaenoides*	4x	AB
***Urvilleana*** ** (A, B)**		
*P. chloroleucum*	4x, 6x?	AB
*P. racemosum*	4x, 6x?	AB(+)
*P. urvilleanum*	4x	AB
***Incertae sedis*** ** (C, D, E)**		
*P. mystasipum*	6x	CDE
*P. olyroides*	4x	CD
***Dichotomiflora*** ** (I, J, K, L)**		
*P. aquaticum*	8x	IJKL
*P. dichotomiflorum*	4x	IL
*P. elephantipes*	6x	KL(+)
*P. gouinii*	4x	JK
*P. pedersenii*	8x	IJKL
*P. aff. aquaticum (CR1, CR3)*	6x, 8x?	IJKL
***Panicum*** ** (F, G, H)**		
*P. bergii*	4x	FG
*P. capillare*	2x	F
*P. miliaceum*	4x	FH
*P. nephelophilum*	2x	F
*P. stramineum*	4x	FG
***Rudgeana*** ** (E)**		
*P. rudgei*	2x	E
*P. cervicatum*	2x	E
*P. campestre*	2x	E
*P. cayennense*	2x	E

Ploidy levels were inferred from sequence types, flow cytometry, and cytological studies. The symbol (+) indicates that other genomic components were suggested in some analyses, but not characterized further in the current study.

### Data Processing, Alignment, Homoeologue Identification, and Sequence Polishing

For the plastid sequences, forward and reverse reads were combined manually, and sequences were aligned in the program MUSCLE 3.52 [70]. Only minimal adjustment was necessary because sequence diversity was low. For nuclear loci, vector sequences and ambiguous bases from the ends of both forward and reverse reads were removed manually. Clone sequences were imported and manually inspected with MEGA version 4 [71]. Ambiguous bases in each clone sequence were corrected manually by comparing sequence quality from trace files. Corrected clones were assembled into individual-specific files and aligned with MUSCLE 3.52; nontarget sequences were detected and removed. For most plants in this study, different sequence types (putative homoeologous loci, presumably duplicated via allopolyploidy) could be visually identified for each gene. Recombinant sequences can arise naturally via homologous recombination or artificially via PCR strand swapping [12], [72], particularly when using a non-proof-reading DNA polymerase. These sequences could be identified by eye through a carefully inspection of the alignment, an approach also used by Brassac et al. [12]. Because these recombinant sequences increase homoplasy and distort the resulting phylogeny, recombinant sequences were excluded. Consensus sequences for each sequence type per individual were constructed to minimize the inclusion of sequencing errors. In general, a substitution that appeared in a single sequence was considered to be PCR error. Sequences with two or more nucleotide differences were interpreted as different alleles. The majority character state was used in the consensus sequence. The sequences were considered to represent different loci only if they clustered in different major clades (A vs. B, etc.). Raw sequences were segregated into homoeologue-specific files, which were aligned with MUSCLE 3.52 and condensed into one consensus sequence for each homoeologue per individual. Consensus sequences from each nuclear locus were then collated into one final file for each locus, which was used for all downstream phylogenetic analyses.

Assembled sequences were used to compile a number of different datasets for each locus, including “full” (containing all available consensus sequences per locus) and “abridged” (containing relevant subsets, as described below and in the Results). Plastid sequences and nuclear consensus sequences were submitted to GenBank (Appendix S1). All plastid and nuclear data matrices are available from the authors upon request.

**Figure 5:**
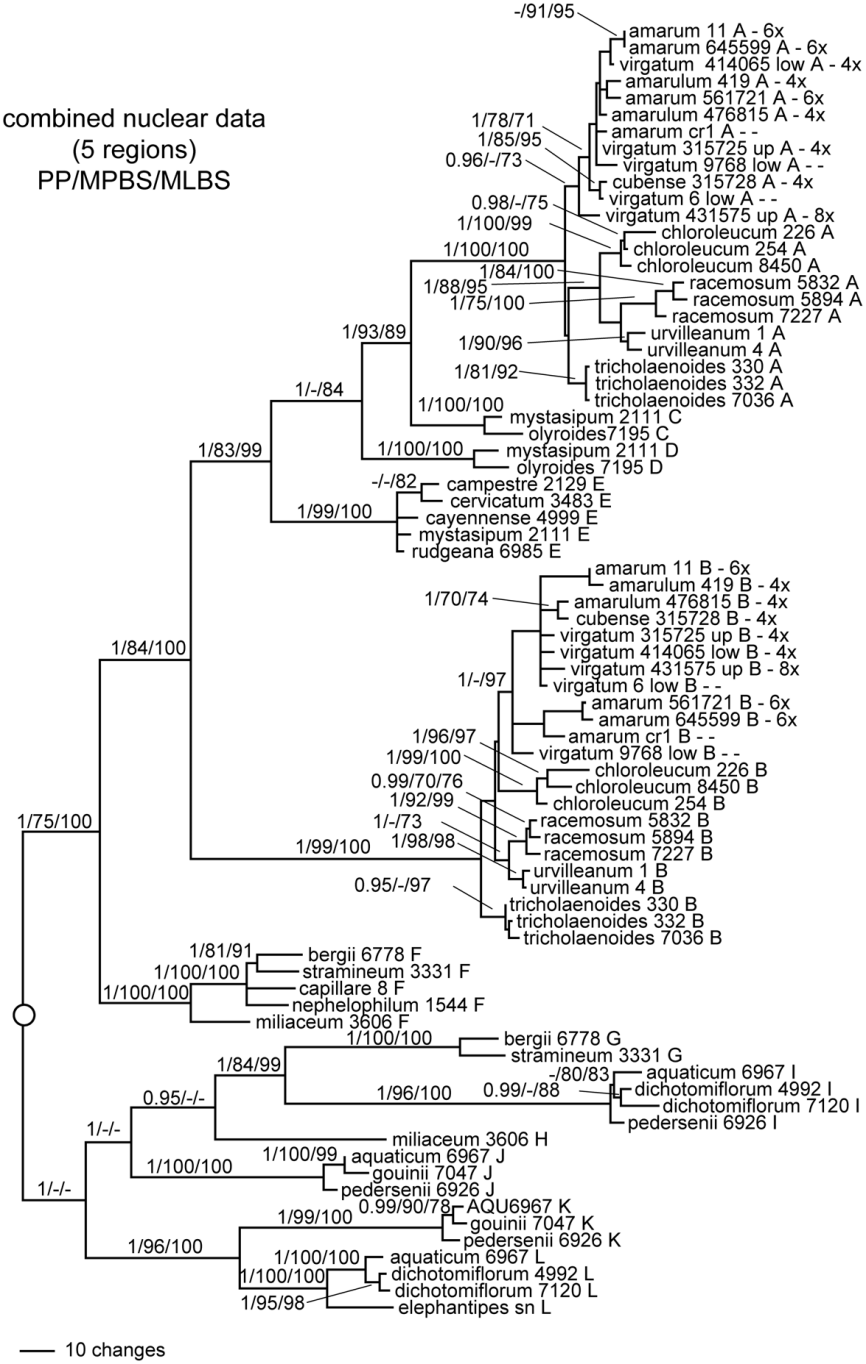
Bayesian phylogram based on combined nDNA data. Support values are Bayesian posterior probability/maximum parsimony bootstrap/maximum likelihood bootstrap. Outgroups have been omitted for clarity.

### Phylogenetic Analyses

Separate analyses were run for each of the seven loci (2 cpDNA regions, 5 nDNA regions). Each data set was analyzed using 1) Bayesian inference (BI) using MrBayes 3.1.2 [73] and 2) parsimony analyses (MP) using PAUP* 4.0b10 [74]. For combined datasets, we also conducted Maximum Likelihood (ML) analyses using GARLI v0.95 [75] with multiple random starting trees. Posterior Probabilities (PP) ≥0.95 and MP and ML bootstrap values ≥70% were recorded on the resulting topologies.

BI analyses were run on GEODE or TOPAZ clusters at the National Museum of Natural History (Smithsonian), and MP and ML analyses were run on a Macintosh G5. For BI and ML analyses, the substitution model for the different data partitions (loci) was determined with a hierarchical likelihood ratio test as implemented in jModelTest [76], using the Akaike Information Criterion (AIC) to select the best model. The number of substitution schemes was set to 7, base frequencies +F, rate variation +I and +G, and the base tree for likelihood calculations was set to ML optimized. A total of 56 models was compared. Models for each data partition are indicated in Table 4. BI and ML analyses of individual data sets were run using the model identified, and combined data sets were run using the most parameter-rich model identified.

For Bayesian phylogenies, four Markov chain Monte Carlo (MCMC) runs were initiated, each with a minimum of 10 000 000 generations (with more generations used when needed to reach stationarity). Prior distribution settings used default values, except for the nucleotide substitution model, which was altered to the general time reversible [GTR] model, and the rate model, which was drawn from a gamma distribution while allowing for invariant sites. Runs were started from a random tree; the topology was sampled every 1 000 geonerations of the MCMC chain. Performance of individual runs was assessed in MrBayes and phylogenies compared between runs. Majority rule (>50%) consensus trees were constructed after removing the first 10% of sampled trees (“burn-in”). Bayesian analyses were highly congruent between runs. Support for clades within BI trees was assessed using posterior probabilities.

For most datasets, MP analyses used 1 000 random addition sequences, tree bisection-reconnection (TBR) branch swapping, and Multrees on. For the three largest datasets (*adh*1, *knotted*1 and *pabp*1 full datasets), parsimony analyses began with 10 000 random addition sequences, TBR branch swapping, and Multrees off to identify multiple islands of trees. Multrees was then turned back on, the maximum number of trees to save (Maxtrees) set to 2 000 000 and the search run to completion. Full heuristic bootstraps were performed for MP with 100 bootstrap replicates.

**Figure 6:**
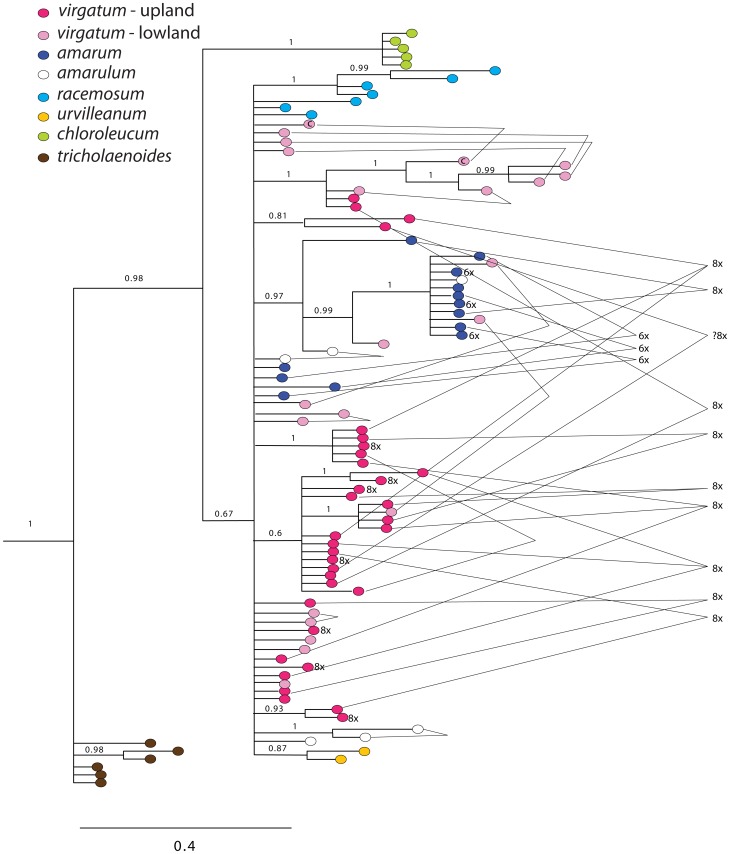
Bayesian phylogram of the analysis of all accessions of sections *Virgata* and *Urvilleana*, using only the B genome of *knotted1*; topology is that presented in Supplemental Figure S1 for the *Virgata*-*Urvilleana* clade, but with sequence names replaced by colored ovals. The pink oval with the letter c indicates sequences from *P*. *virgatum* var. *cubense*. Unlabeled ovals represent sequences from tetraploids. Ovals labeled with 6x or 8x indicate hexaploid or octoploid plants, respectively, from which only one sequence type was recovered. Slender lines connect sequences from the same plant. Vertices labeled 6x or 8x indicate hexaploid or octoploid plants respectively; unlabeled vertices indicate tetraploids. Numbers above branches are Bayesian posterior probabilities.

**Figure 7:**
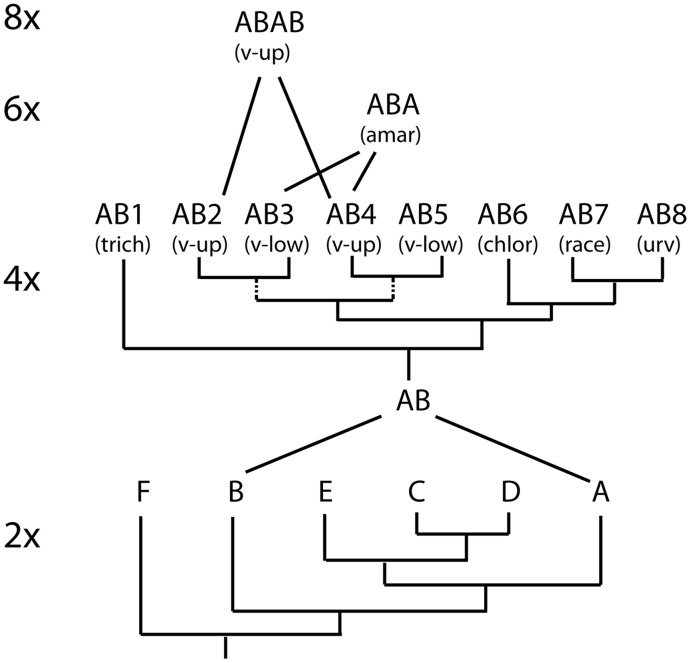
Cartoon of relationships among the species of sections *Virgata* and *Urvilleana* and their close relatives. Diversification occurred at the diploid level, a hybridization event involving a seed parent with the A genome and a pollen parent with the B genome gave rise to an allotetraploid offspring. Diversification then occurred at the tetraploid level. An AB genome tetraploid crossed with another AB tetraploid to give rise to octoploids and hexaploids. Octoploid *P. amarum* is omitted for clarity.

Maximum likelihood (ML) topology and branch support was estimated using GARLI based on 100 bootstrap replicates, with runs set for an unlimited number of generations, and automatic termination following 10 000 generations without a significant topology change (lnL increase of 0.01).

For the two chloroplast datasets and the five abridged nuclear datasets respectively, we used the incongruence length difference test (ILD) [77] as implemented in PAUP* to examine topological incongruence among the data sets. These two analyses applied 100 test replicates, each with 100 random order entry heuristic searches and one tree saved per replicate.

For nuclear loci, we first analyzed all available consensus sequences (“unabridged datasets”). We then used a number of different abridged and/or concatenated data sets to address specific questions. For example, sequences from individuals of *P. virgatum* fell into two divergent clades (**A** and **B**), with each individual having at least one **A** and one **B** sequence type (putative homoeologues, a result that is consistent with allotetraploidy, where each sequence type represents a different parental lineage). Higher order polyploids had more than one of each type, as expected (e.g., **A1**, **A2**, **B1**, **B2**), indicating subsequent hybridization events. However, these higher order polyploids occurred only in the *virgatum*/*amarum*/*amarulum* clade. In order to investigate the position of *virgatum*/*amarum*/*amarulum* with respect to all other taxa in the analysis, we did some analyses in which we assembled an abridged data set for each of the five nuclear regions, using only 1 sequence per subtype (e.g., **A1** and **B1**) per individual. This approach over-simplifies species-level relationships within *virgatum*/*amarum*/*amarulum*, but is useful for exploring relationships between the two major clades (**A** and **B**). Five abridged datasets were combined to create a concatenated nuclear data set, with 5 nDNA regions and 83 taxa. Lastly, for species in sections *Urvilleana* and *Virgata*, we also explored a number of different data concatenations, combining data from different loci and different homoeologues (**A** and **B**), each of which provides independent estimates of phylogeny. Additional methodological details of these data explorations are included in the Results, as relevant.

We also tested whether data provided sufficient evidence to reject alternative hypotheses of relationships, including different explanations for the origin of multiple gene copies in the nuclear genome and the monophyly of sections *Urvilleana* and *Virgata*. For each hypothesis, constraint trees were constructed in MacClade 4.08 [78]. Shimodaira-Hasegawa (SH) [79], Kishino-Hasegawa (KH) [80], and Shimodaira Approximately Unbiased (AU) [81] tests, as implemented in PAUP*, were used to test the significance of differences in tree statistics amongst different topologies.

## Results

### Flow Cytometry

Flow cytometry estimates of ploidy are listed in Table 3, and appended to labels in the figures. In all cases, standards yielded the expected patterns of relative fluorescence intensity, and sampled individuals were easily categorized as tetraploid (*P. amarulum*, *P. virgatum*), hexaploid (*P. amarum, P.* aff. *aquaticum*), or octoploid (*P. amarum*, *P. virgatum*).

### Phylogenetic Analyses: Chloroplast Regions

Chloroplast sequence data characteristics are summarized in Table 4. Separate analyses of the two cpDNA datasets indicated no hard incongruence (ILD test: P value  = 0.73), so these datasets were combined. The resulting data matrix included 3222 characters, of which 128 were parsimony informative. Results were congruent across different methods of analysis. The Bayesian phylogram for the combined cpDNA data is presented in Fig. 1. The MP analysis resulted in 66 equally parsimonious trees with a length of 323 (CI = 0.8066, excluding autapomorphies).

*Panicum s. s.* was resolved into at least two clades (Fig. 1), one including all representatives of sections *Panicum* and *Dichotomiflora* (1.0 PP, 96% MP, 83% ML), and the other all representatives of sections *Rudgeana*, *Virgata*, and *Urvilleana* (−, 70, 86). *Panicum elephantipes* and *P. gouinii* (section *Dichotomiflora*) were on relatively long branches, forming a weak cluster in an unresolved polytomy with the remaining taxa in sections *Dichotomiflora* and *Panicum*. *Panicum aquaticum*, *P. dichotomiflora*, and *P. pedersenii* (also sect. *Dichotomiflora*) formed a clade with robust support (1.0, 100, 100) but little internal resolution. Both samples of *Panicum* aff. *aquaticum* from Costa Rica were nested in this clade, but were not identical to each other nor to any other individual in the clade. Section *Panicum* received moderate to strong support (1.0, 86, 85); internal branching suggested a relationship between *P. bergii* and *P. stramineum* (0.97 PP, but weak bootstrap support).

The other major clade consisted of robust subclades for (1) section *Urvilleana* + section *Virgata*, hereafter called the VU clade, (2) section *Rudgeana*, and (3) *P. mystasipum* + *P. olyroides*. *Panicum mystasipum* and *P. olyroides* were sister species (1.0, 97, 97), and these two formed a moderately-supported clade with *Urvilleana* + *Virgata* (1.0, 82, <50), which was sister in turn to section *Rudgeana* but with weak support (70% MPB). The monophyly of section *Rudgeana* was strongly supported (1.0, 99, 100) within which *P. campestre* and *P. rudgei* were sister species (1.0, 100, 100).

The VU clade (sections *Urvilleana* and *Virgata*) received robust support (1.0, 100, 100), although species-level resolution within the clade was generally poor. Sequences from *P. tricholaenoides* (sect. *Virgata*) formed a robust subclade (1.0, 94, 85). All individuals of *P. chloroleucum*, *P. racemosum*, and *P. urvilleanum* (all sect. *Urvilleana*) formed a weak clade. Both *P. chloroleucum* and *P. racemosum* had more than one cpDNA haplotype.

All lowland ecotypes of *P. virgatum* (all 4x) formed a distinct clade (1.0, 95, <50), including four cpDNA haplotypes: 1) accessions from Arkansas, Kansas, and Texas, 2) two samples from North Carolina, 3) all remaining samples of lowland switchgrass from Maryland, Florida, and Mexico, and 4) *P. virgatum* subsp. *cubense*. The lowland *virgatum* clade was no more closely related to upland *virgatum* than to either *P*. *tricholaenoides* or *P*. sect. *Urvilleanum*.

All but one haplotype of upland *P. virgatum* (4x and 8x), *P. amarum* (4x, 6x, 8x), and *P. amarulum* (4x) formed a weak group (0.95 PP). Within this cluster, several distinct haplotypes were identified for upland ecotypes of *P. virgatum*, and for *P. amarum* + *P. amarulum*. One upland haplotype was recovered from both 4x and 8x individuals, while the other haplotypes were only found in octoploids. One *amarum* haplotype encompassed individuals of *P. amarum* and *P. amarulum*, with ploidy ranging from 4x to 8x. The remaining *amarum* haplotype was recovered from hexaploid individuals of *P. amarum* from North Carolina and Louisiana, plus one Mexican sample of unknown ploidy. One upland *virgatum* haplotype recovered from three individuals (315724, 476292, and 642193) formed a weak sister relationship with the *amarum* haplotypes. A single accession of *P. virgatum* (414066, 8x, upland, New Mexico) was distinct and not clearly related to other haplotypes of *P. virgatum.*

### Phylogenetic Analyses: Nuclear Regions

#### 1. Analyses of separate nDNA regions

Nuclear sequence data characteristics are summarized in Table 4. Data sets for all five nuclear regions included representatives of all species (except *P.* aff. *aquaticum* in the two cellulase datasets), plus different cytotypes of *P. virgatum* and *P. amarum*, and different ecotypes of *P. virgatum*. However, we did not attempt to retrieve all loci for all sampled individuals (e.g., 33 individuals of *P. virgatum* are represented in *knotted*1 vs. five individuals of *P. virgatum* in *PvCel*1). The BI phylogram of an analysis of the full dataset for one representative region (*adh1*) is presented in Figure 2, and the abridged dataset for another (*knotted*1) is presented in Fig. 3, along with summaries of all five regions in Fig. 4. (Complete phylograms for the remaining regions are available as Supplemental Figures S1, S2, S3, S4 [unabridged data sets] and S5, S56, S7, S8 [abridged data sets]).

We found robust support for 12 major clades, and taxon membership in these clades was similar among the five nuclear genes (Table 5); for clarity, we here designate these clades by the letters **A**–**L**. For example, a clade containing all sampled representatives of *Panicum* section *Rudgeana* (**E**) was recovered in analyses of all five loci, while two non-sister clades (**A** and **B**) were recovered for all representatives of sections *Virgata* and *Urvilleana*.

For many individuals, cloning recovered multiple, strongly divergent sequences types (homeologues) for each sampled locus (Figs. 2, 3). In general, the number of sequence types recovered from a given individual correlated with ploidy level: a single sequence type was recovered from diploids, two from tetraploids, three from hexaploids, and four from octoploids. Because no signs of duplication were found in diploids, we assume that multiple sequence types in polyploids correspond to duplication via hybridization and allopolyploidy. In most cases, the different sequence types were strongly divergent and nested in different major clades. For example, the tetraploid *P. bergei* had two sequence types that fell in clades **F** and **G** respectively, while the octoploid *P. aquaticum* was represented by sequences in clades **I**, **J**, **K** and **L** (Figs. 2, 3, Table 6).

Because we were focusing particularly on sections *Virgata* and *Urvilleana*, we did not attempt to recover all major lineages for species in sects. *Panicum* or *Dichotomiflora*. For example, we did not retrieve a *knotted*1 sequence from *P. miliaceum* falling into lineage **H**, nor a sequence of *PvCel*2 from *P. pedersenii* in lineage **I**. We expect that these loci are present, but were simply missed by our amplification and cloning strategy. Relationships among these clades varied among trees.

Each sampled species and individual from sections *Urvilleana* and *Virgata* (including *P. virgatum*) had at least two distinct sequence types (homeologues), which fell into clades **A** and **B**. Hexaploid and octoploid individuals had more than one sequence type per major clade (e.g., some hexaploid individuals of *P. amarum* had two **B** clade sequences in *knotted1*, Supplemental Fig. S1).

Individuals in sect. *Rudgeana* are presumed to be diploid based on published chromosome counts, and in every case only one sequence type was recovered per individual per region. These form a clade that we have designated **E**. *Panicum olyroides* (4x; [82]) has two distinct sequence types that are closely related to lineages **A**, **B**, and **E**, but not nested within any one of these. The two lineages are thus designated as **C** and **D**. *Panicum mystasipum* has **C** and **D** sequence types like *P. olyroides* plus **E**-type sequences from sect. *Rudgeana*, suggesting that the sampled individual is hexaploid. Note that among the five regions, we arbitrarily assigned the name **A** to the clade that was most closely related to clades **C**, **D**, and **E**.

Two diploids occur in lineage **F** (*P. capillare* and *P. nephelophilum*). Tetraploids in this lineage are composites of more than one lineage. For example, *P. miliaceum* has sequences from clades **F** and **H**, while *P. stramineum* and *P. bergii* are **FG**. Of the remaining lineages, lineage **G** is represented only by *P. stramineum* and *P. bergii*. None of the sampled diploids were associated with this clade. The other putative parent lineage of *P. miliaceum* is labeled **H**, but no diploid was sampled from this lineage. Thus, one of the parents of *P. miliaceum* may be similar to diploids *P. capillare* and *P. nephilophilum*. *Panicum dichotomiflora* and allies in section *Dichotomiflora* are here represented by several distinct lineages that do not form a monophyletic group. The constituent lineages are identified as **I** (represented by *P. aquaticum, P. dichotomiflora,* and *P. pedersenii*), **J** (represented by *P. aquaticum, P. gouinii*, and *P. pedersenii*), **K** (represented by *P. aquaticum, P. elephantipes, P. gouinii*, and *P. pedersenii*), and **L** (represented by *P. aquaticum, P. dichotomiflora, P. elephantipes*, and *P. pedersenii*). None of the sampled taxa from this section are diploids, and the polyploids all consist of combinations of different lineages. For example, *P. dichotomiflorum* is **IL**, *P. gouinii* is **JK**. *Panicum aquaticum* and *P. pedersenii* had sequences from at least four different lineages (**IJKL**). Both *P. pedersenii* and *P. aquaticum* may be related to tetraploids such as *P. dichotomiflora* and *P. gouinii*, which are both composites of multiple lineages (**IL** and **JK**, respectively).

Each nDNA region provided some unique information regarding relationships (Figs. 2, 3, 4, S1, S2, S3, S4, S5, S6, S7, S8). For example, in the *adh*1 topology, lineage **F** (represented by sequences recovered from *P. miliaceum*, *P. capillare*, *P. bergii*, *P. stramineum*, and *P. nephilophilum*) was sister to **ABCDE** with robust support (96% BS). The other nuclear topologies neither supported nor conflicted with this relationship, but were simply unresolved. The *adh*1 region also revealed more sequence types among species in lineages **A** and **B**, possibly indicating lineage-specific gene duplications (Figs. 2, 4). Moreover, we found an unusual lineage of *adh*1 and *PvCel*2 in *P. racemosum*, close to the **C** genome (possibly consistent with gene duplication unique to this species).

In regions *PvCel*2, *knotted*1, and *pabp*1, three distinct sequence types were found for *P. elephantipes* associated with **K** and **L** clades. Some of these sequences were labeled “**kl**” in some figures (S1, S2, S4, S6, S8), to indicate uncertain placement.

#### 2. Combined analysis of nDNA data – *Panicum s.s*

All nuclear genes identified the same major lineages (**A**–**L**), although the relationships among them were generally poorly supported in any single data set and generally differed between data sets (Fig. 4). The partition homogeneity test indicated weak incongruence among the five nuclear data sets (ILD: *P* = 0.0333), but we interpret this as a result of lack of resolution rather than hard incongruence. Accordingly, we attempted to improve phylogenetic resolution among the major lineages by assembling a combined nDNA dataset, using plants for which we had data from all five loci.

The combined dataset included 83 sequences and consisted of a total of 4388 bp (aligned). The branching order amongst lineages **ABCDE** received robust support (Fig. 5), with the two constituents of *Virgata*/*Urvilleana* (**A** and **B**) as non-sister clades, indicating that the VU clade is the product of a wide cross followed by allopolyploidy. Forcing **A** and **B** sequences to be sister clades was rejected by the SH, AU, and KH tests (p<0.05, p<0.05, and p<0.0001, respectively).

The combined analysis indicated that lineage **F** (including sequences from species in *Panicum* sect. *Panicum*) is sister to the group **ABCDE** (1.0, 75, 100), while the other sequences from *Panicum* sect. *Panicum* (clade **G**) is sister to lineage **I** (1.0, 84, 99). The combined data also strongly support **K** and **L** as sister lineages (1.0, 96, 100), as indicated by all loci except *knotted*1 (Figs. 3 and 4). Clade **H** (*P. miliaceum*) showed a weak association with **G** and **I** (0.95, <50, <50), while **J** (a component of species in sect. *Dichotomiflora*) was indicated to be sister to **H+GI** (1.0, <50, <50).

Within lineages **A** and **B**, the combined analysis was inconclusive regarding the monophyly of sections *Urvilleana* and *Virgata*. Neither the SH test, the KH test, nor the AU test was able to reject monophyly of section *Virgata*. Within section *Urvilleana*, *P. racemosum* and *P. urvilleanum* showed a close relationship in both the **A** and **B** clades. *Panicum tricholaenoides* (section *Virgata*) was associated with sections *Virgata* and *Urvilleana*, but distinct from species in either section. No clear relationship emerged among species in the switchgrass complex (*P. amarum*, *P. amarulum*, and ecotypes of *P. virgatum*).

#### 3. Higher order polyploids – Sections *Virgata* + *Urvilleana*

The analyses described above do not resolve relationships among the ecotypes of *P. virgatum* and the closely related *P. amarum* and *P. amarulum*, in part due to incomplete sampling of alleles and inclusion of a limited number of accessions. To dissect the history of the hexaploid and octoploid species in the switchgrass complex, we therefore focused on data from *knotted1*, for which we had samples of more individuals. Figure 6 shows the phylogeny of the VU clade based on only the **B** genome sequences of *knotted*1. This represents sequences from a single genome of the two present in the tetraploids; we expect two **B** genome copies in octoploids, and one or two in hexaploids depending on their origin.

As shown in Figure 6, most tetraploids had one sequence type, but a few had two sequence types distinguished by a few mutations. For example, the sample of *P. virgatum* var. *cubense* (indicated by pink symbols labeled with a c, joined by slender lines) had two sequences that were distinct but not strongly dissimilar. Likewise, two of the individuals of *P. amarulum* (white symbols, joined by slender lines) were each represented by two quite similar sequence types. However, in a few of the tetraploids, we discovered quite different sequences suggesting unusually high heterozygosity at the *knotted1* locus of the **B** genome.

Sequences from the hexaploid *P. amarum* mostly were in a clade with a few sequences from lowland *P. virgatum*, although a few fell in an unresolved position at the base of the tree. Although the chloroplast data indicate that *P. amarum* has an upland *P*. *virgatum* cytoplasm, we did not see evidence of upland *knotted1* alleles clustering with *P*. *amarum* sequences.

Octoploid *P. virgatum*, which is morphologically classified as the upland ecotype, appears to be an allopolyploid formed from disparate ancestors. We expected two distinct B genome sequences in the octoploid individuals, but for several of them we found three sequences, suggesting that the tetraploid ancestors that formed the octoploid were themselves heterozygous. Sequences in the octoploids for which we recovered more than one sequence type were dissimilar, and generally fell into distinct, well-supported clades. The octoploids thus appear to be allopolypoids, formed by wide crosses.

## Discussion

### Allopolyploid Speciation in *Panicum* and Origin of the A and B Genomes

All five nuclear gene tree topologies identified the same major clades, here designated as **A** through **L**. The **A** and **B** lineages clearly correspond to the two distinct genomes identified by Okada et al. [35] for tetraploid *Panicum virgatum*. By analogy, we surmise that the other ten lineages are also genomic groups, although this would need to be confirmed by cytogenetic evidence. Putative genomic formulas are summarized in Table 5.

*Panicum* sections *Virgata* and *Urvilleana* (the VU clade) together are the result of a single polyploidization event combining the **A** and **B** genomes; this result is supported by all five nuclear loci. Because the relationships of the **A** genome are similar to those found in the chloroplast data set, we infer that the seed parent provided the **A** genome in original cross. (Maternal inheritance of the chloroplast has been experimentally verified in switchgrass [34]). Following this ancestral hybridization event, diversification occurred at the tetraploid level (Figure 7) to form ∼15 nominal species currently classified in the two sections, *Virgata* and *Urvilleana*. Thus any individual plant of any member of the VU clade has at least two copies of each nuclear gene – one representing the **A** genome and one representing the **B** genome – and appears at least twice in the gene tree.

The diploids most closely related to the VU clade are in sect. *Rudgeana*. We have sampled four (*P. rudgei*, *P. campestre*, *P. cayennense*, *P. cervicatum*) of the six species in the section, which is native to Central and South America [24]. This section may also contain the African *P. congoense* (M. Estep and E. Kellogg, unpubl.). Each species had only one sequence type, consistent with the reported ploidy level; allelic variation was essentially non-existent, or could not be distinguished from PCR error. The section is monophyletic in all our analyses, consistent with its distinctive morphological characters, including an unusual rachilla structure beneath the upper floret. This group may provide the best model for the diploid progenitor of the **A** genome, and efforts should be made to obtain seeds or other living material for comparison to switchgrass.

Closely related to sects. *Virgata*, *Urvilleana* and *Rudgeana* are *P. olyroides* and *P*. *mystasipum*, previously placed incertae sedis [24]. *Panicum olyroides* is an apparent allotetraploid hybrid between unknown diploids from lineages **C** and **D**, while *P. mystasipum* is apparently an allohexaploid hybrid between *P. olyroides* and a diploid from sect. *Rudgeana*. The **A** genome is related to at least one of the genomes in *P. mystasipum* and *P. olyroides*.

The **B** genome appears only in polyploids and is sister to the ancestor of the **A**, **C**, **D**, and **E** genomes. We have not yet sampled any diploids that fall into the **B** lineage; these may be extinct or may be among the remaining ca. 75 species of *Panicum* not included here.

Sections *Panicum* and *Dichotomiflora* (genomes **F** through **L**) share a complex history of hybridization and polyploidization. Because they were not the focus of this study, we have not attempted to disentangle the relationships among them. We have shown, however, that *Panicum miliaceum*, common millet, is clearly formed from a tetraploidy event separate from the one that led to sects. *Virgata* and *Urvilleana*.

Among all the allopolyploids in *Panicum*, regardless of the gene region investigated, parental genomes are still identifiable (e.g., **A** and **B**). We see little evidence of rapid gene loss, as has been described for other polyploidization events [83], [84]. Our data also refute the suggestion of Missaoui et al. [44] that switchgrass is an autotetraploid.

### Relationships within the VU Clade

The five nuclear and two chloroplast gene trees support a close relationship of species in sections *Virgata* and *Urvilleana*. In most individual gene trees, sequences for *P. tricholaenoides* coalesce within the species, which is sister to all other members of the clade. In general, we found distinct clades made up of most or all of the sequences of *P. racemosum*, *P. urvilleanum*, *P. virgatum* upland, *P. virgatum* lowland, and *P. amarum*. In the combined nuclear gene tree, *P*. *racemosum* and *P*. *urvilleanum* are sisters; *P*. *chloroleucum* is sister to the *racemosum*/*urvilleanum* clade in the A genome, but not the B genome sequences.

Lowland and upland *P. virgatum*, *P. amarum*, and *P. amarulum* together form a well-supported clade but are only weakly differentiated among themselves. *P. amarum*, *P. virgatum* lowland ecotypes, and *P. virgatum* upland ecotypes represent three distinct chloroplast (maternal) lineages. The nuclear phylogeny, in contrast, suggests some intermingling of gene pools (Figs. 5, 6).

Some presumed tetraploid individuals (e.g., *virgatum* 431575) have 2 A-type sequences and 2 B-types (not shown). While often these sequences are sisters in gene trees, suggesting that they are simply allelic variants, in some cases they assemble into subclades (without strong support) that do not track species. For example, a tetraploid individual of *P. virgatum* may have one allelic form of **A** that is more similar to alleles in *P. amarum*, and another that is more similar to *P. virgatum*. This suggests hybridization or incomplete lineage sorting or both. Switchgrass is predominantly outcrossing, and ongoing hybridization is likely.

### Origin of Hexaploids in Switchgrass and Its Closest Relatives

Figure 6 shows that hexaploid and octoploid plants are themselves formed from divergent lineages of tetraploids, and thus that the higher order polyploids are allopolyploids. *Panicum amarum* hexaploids (and the one octoploid) have nuclear genes similar to those in lowland *P. virgatum*, even though they have upland cytoplasm. This conclusion needs to be tested with a much broader sample of plants from regions where they co-occur.

The hexaploids may have formed either from a cross between an octoploid and a tetraploid, or from a cross between two tetraploids with one parent providing a reduced and the other an unreduced gamete. Our data point to the latter hypothesis, in that we did not find any evidence of alleles shared between the hexaploids and octoploids. In either case, chromosome pairing should be disrupted. This would explain the sterility described by Palmer [54] for *P. amarum*. She noted a high number of trivalents as well as other problems during meiosis, as would be expected.

The data on higher-level polyploids also provide some insight into classification. Plants in this study that are morphologically classified as *P*. *amarum* are hexaploid (except for ama7, a plant from North Carolina), while those classified as *P. amarulum* are tetraploid. Once again our sample size is not large enough to be definitive, but we suggest that *P. amarulum* may not be a distinct entity, but rather represents lowland *P*. *virgatum* with possible introgression from *P. amarum*. Some of the nuclear loci point to a particularly complex history for the octoploid plant of *P. amarum* (ama7)(Fig. 2), but the plant is morphologically unremarkable.

We investigated 13 octoploid accessions of *P. virgatum*, which are morphologically and cytoplasmically classified as representing the upland ecotype. Our data are consistent with the hypothesis that the ancestors of the octoploids were themselves upland, but we cannot rule out the possibility of admixture of some genes from lowland ecotypes.

### Conclusions

In *Panicum* s. s., hybridization and polyploidy have been and continue to be important evolutionary forces. The current study documents the prevalence of allopolyploids in *Panicum* s. s., with 17 of 23 sampled species showing evidence of allopolyploid origins. Moreover, we show that allopolyploidy is not a dead end, but potentially a fast track for ongoing diversification.

As summarized in the schematic diagram in Figure 7, *Panicum* apparently experienced a period of cladogenesis, leading to the lineages we have called **A** through **L**. These lineages subsequently came together via hybridization, and then diversified again to produce new species within allopolyploid lineages. These new tetraploids then came together via hybridization and polyploidy, generating yet more morphological diversity.

Our data only hint at the complex morphological and genomic relationships in the *Panicum virgatum* complex. Additional studies in *P. virgatum* and *P. amarum*, with more accessions and additional phylogeographic data, are necessary to unravel their historical relationships and current genetic connections. Such groups of related polyploids offer the possibility of testing whether polyploids really are more ecologically or phenotypically diverse than their diploid parents, and whether they radiate into new niches even in the short term.

## Supporting Information

Figure S1 *knotted1* dataset, unabridged. Phylogram from Bayesian analyses; numbers above branches indicate posterior probabilities above 0.5. Taxon labels are in the format: B_k1VIR421901_B1.3 where B_ indicates that the sequence belongs to the B genome; k1  =  *knotted1*; VIR421901  =  *P. virgatum* (PI 421901); and B1.3 indicates sequence type B1, for which we recovered 3 clones.
(TIF)

Figure S2 *pabp1* dataset, unabridged. Phylogram from Bayesian analyses; numbers above branches indicate posterior probabilities above 0.5. Taxon labels are in the format: B_p1AMA11_B1.3 where B_ indicates that the sequence belongs to the B genome; p1  =  *pabp1*; AMA11  =  *P. amarum* (Youngstrom 11); and B1.3 indicates sequence type B1, for which we recovered 3 clones.
(TIF)

Figure S3 *PvCel1* dataset, unabridged. Phylogram from Bayesian analyses; numbers above branches indicate posterior probabilities above 0.5. Taxon labels are in the format: B_c1CHL226_B2.2 where B_ indicates that the sequence belongs to the B genome; c1  =  *PvCel1*; CHL226  =  *P. chloroleucum* (Cialdella 226); and B2.2 indicates sequence type B2, for which we recovered 2 clones.
(TIF)

Figure S4 *PvCel2* dataset, unabridged. Phylogram from Bayesian analyses; numbers above branches indicate posterior probabilities above 0.5. Taxon labels are in the format: B_c2CHL226_B1.1 where B_ indicates that the sequence belongs to the B genome; c2  =  *PvCel2*; CHL226  = *P. chloroleucum* (Cialdella 226); and B1.1 indicates sequence type B1, for which we recovered 1 clone.
(TIF)

Figure S5 *adh1* dataset, abridged to include fewer sequences from *P. virgatum*, *P. amarum*, and *P. amarulum*. Phylogram from Bayesian analyses; numbers above branches indicate posterior probabilities above 0.5. Taxon labels are in the format: B_a1CHL226_B2.1 where B_ indicates that the sequence belongs to the B genome; a1  =  *adh1*; CHL226  = *P. chloroleucum* (Cialdella 226); and B2.1 indicates sequence type B2, for which we recovered 1 clone.
(TIF)

Figure S6 *pabp1* dataset, abridged to include fewer sequences from *P. virgatum*, *P. amarum*, and *P. amarulum*. Phylogram from Bayesian analyses; numbers above branches indicate posterior probabilities above 0.5. Taxon labels are in the format: B_p1AMA11_B2.4 where B_ indicates that the sequence belongs to the B genome; p1  =  *pabp1*; AMA11  =  *P. amarum* (Youngstrom 11); and B2.4 indicates sequence type B2, for which we recovered 4 clones.
(TIF)

Figure S7 *PvCel1* dataset, abridged to include fewer sequences from *P. virgatum*, *P. amarum*, and *P. amarulum*. Phylogram from Bayesian analyses; numbers above branches indicate posterior probabilities above 0.5. Taxon labels are in the format: B_c1AMA11_B2.1 where B_ indicates that the sequence belongs to the B genome; c1  =  *PvCel1*; AMA11  = *P. amarum* (Youngstrom 11); and B2.1 indicates sequence type B2, for which we recovered 1 clone.
(TIF)

Figure S8 *PvCel2* dataset, abridged to include fewer sequences from *P. virgatum*, *P. amarum*, and *P. amarulum*. Phylogram from Bayesian analyses; numbers above branches indicate posterior probabilities above 0.5. Taxon labels are in the format: B_c2CHL254_B1.2 where B_ indicates that the sequence belongs to the B genome; c2  =  *PvCel2*; CHL254  =  *P. chloroleucum* (Cialdella 254); and B1.2 indicates sequence type B1, for which we recovered 2 clones.
(TIF)

Appendix S1 List of species, abbreviated name, voucher number, herbarium, and GenBank accession numbers. Specimens with PI numbers are listed in the herbarium database with Kellogg as collector. Material at UM-St. Louis is indicated by the abbreviation UMSL.
(DOCX)
