# Supplementary figures and images for: Five Nuclear Loci Resolve the Polyploid History of Switchgrass (Panicum virgatum L.) and Relatives
Source: PLoS One. 2012 Jun 18;7(6):e38702. doi: 10.1371/journal.pone.0038702 (PMC3377691; doi:10.1371/journal.pone.0038702)

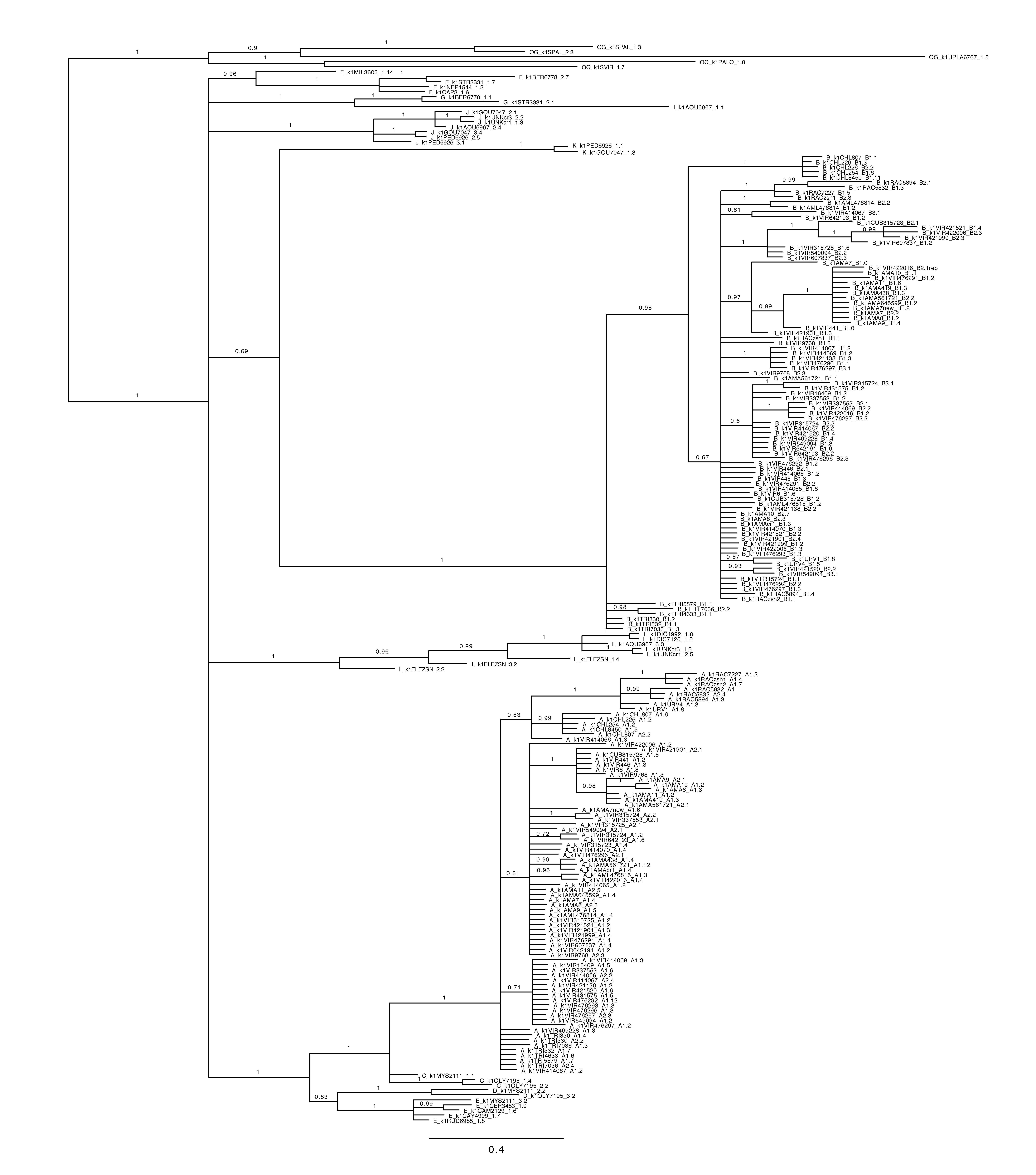

Supplement: Figure S1 — knotted1 dataset, unabridged. Phylogram from Bayesian analyses; numbers above branches indicate posterior probabilities above 0.5. Taxon labels are in the format: B_k1VIR421901_B1.3 where B_ indicates that the sequence belongs to the B genome; k1 = knotted1; VIR421901 = P. virgatum (PI 421901); and B1.3 indicates sequence type B1, for which we recovered 3 clones. (TIF) [file pone.0038702.s001.tif]

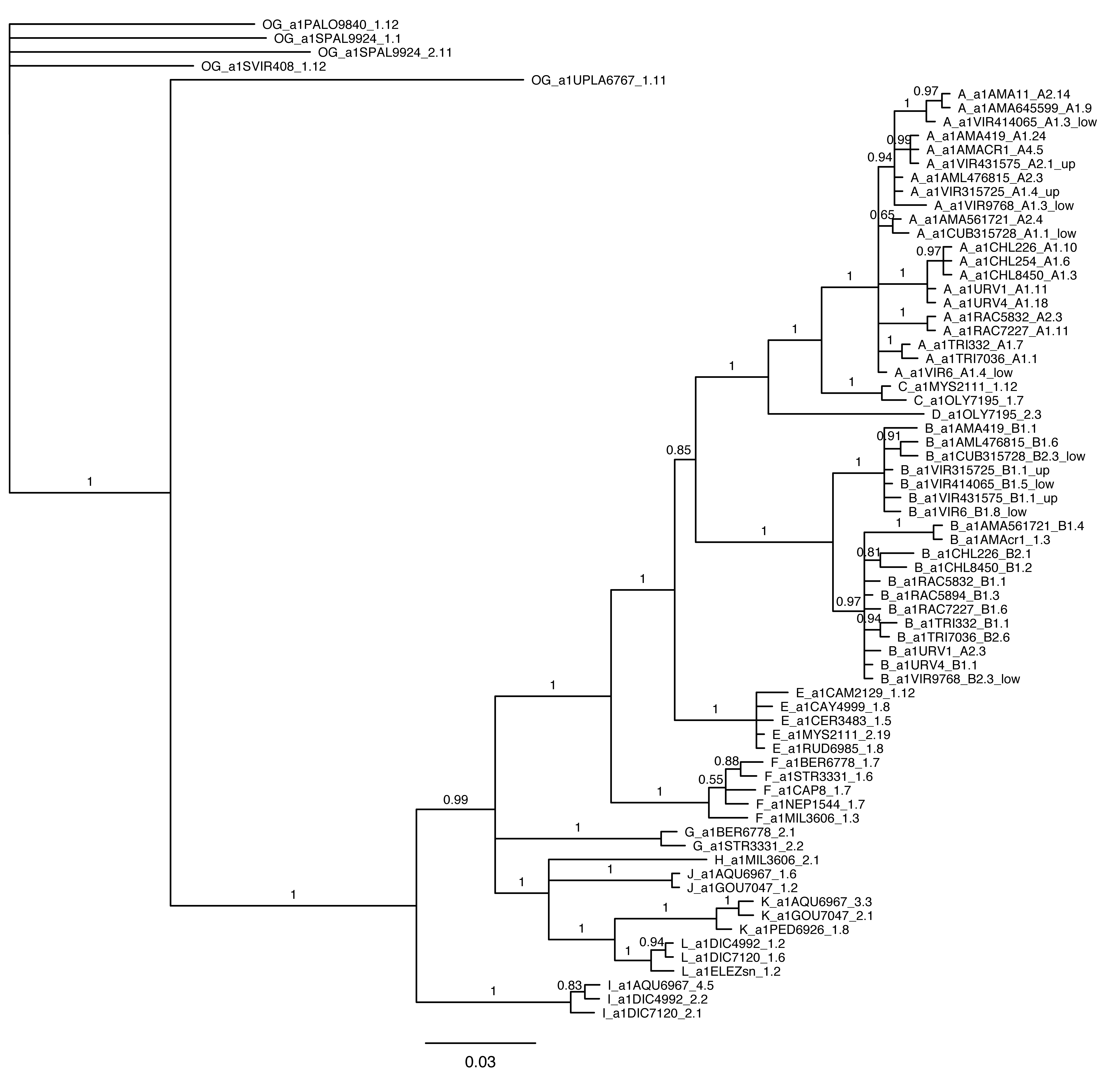

Supplement: Figure S5 — adh1 dataset, abridged to include fewer sequences from P. virgatum, P. amarum, and P. amarulum. Phylogram from Bayesian analyses; numbers above branches indicate posterior probabilities above 0.5. Taxon labels are in the format: B_a1CHL226_B2.1 where B_ indicates that the sequence belongs to the B genome; a1 = adh1; CHL226 = P. chloroleucum (Cialdella 226); and B2.1 indicates sequence type B2, for which we recovered 1 clone. (TIF) [file pone.0038702.s005.tif]

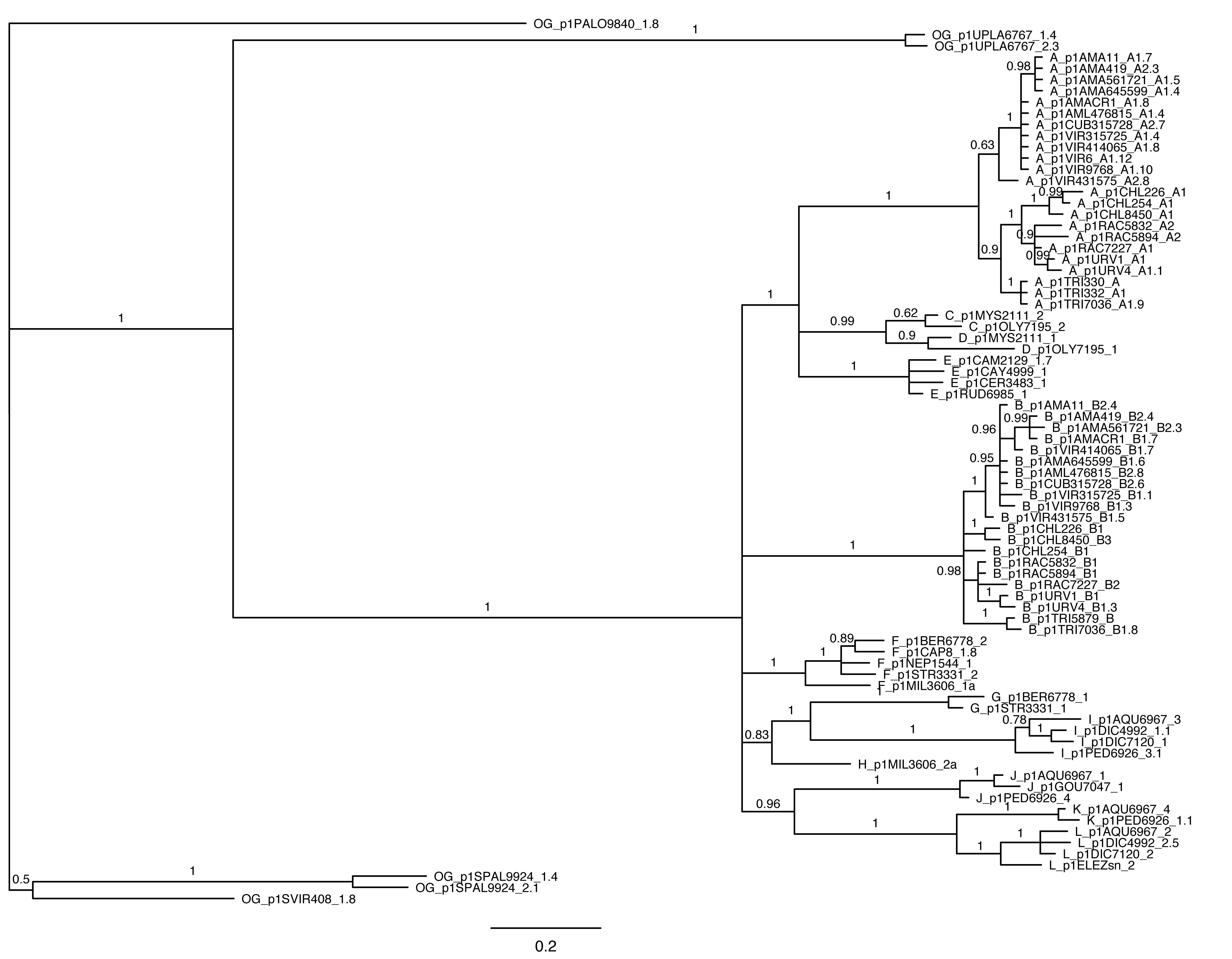

Supplement: Figure S6 — pabp1 dataset, abridged to include fewer sequences from P. virgatum, P. amarum, and P. amarulum. Phylogram from Bayesian analyses; numbers above branches indicate posterior probabilities above 0.5. Taxon labels are in the format: B_p1AMA11_B2.4 where B_ indicates that the sequence belongs to the B genome; p1 = pabp1; AMA11 = P. amarum (Youngstrom 11); and B2.4 indicates sequence type B2, for which we recovered 4 clones. (TIF) [file pone.0038702.s006.tif]

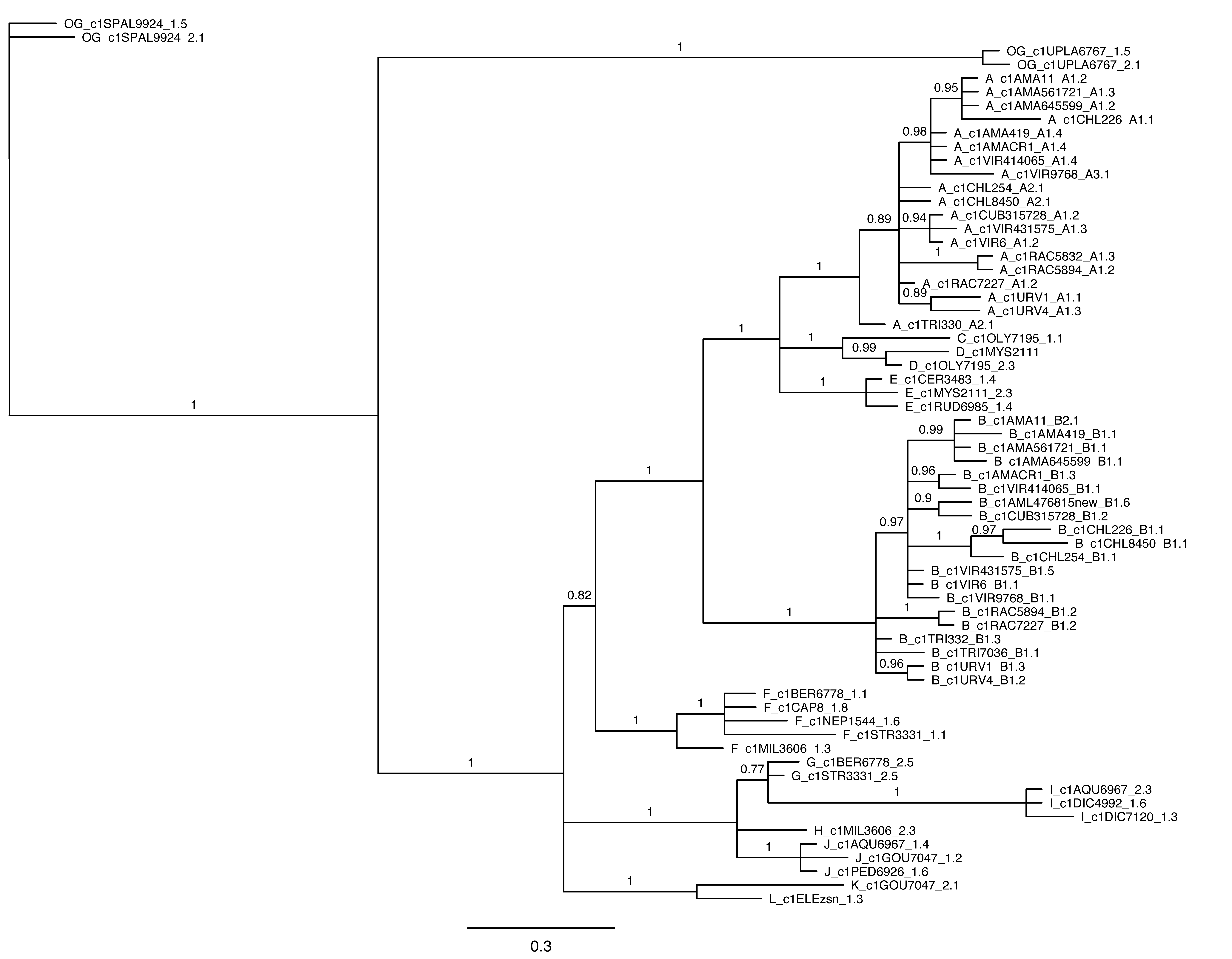

Supplement: Figure S7 — PvCel1 dataset, abridged to include fewer sequences from P. virgatum, P. amarum, and P. amarulum. Phylogram from Bayesian analyses; numbers above branches indicate posterior probabilities above 0.5. Taxon labels are in the format: B_c1AMA11_B2.1 where B_ indicates that the sequence belongs to the B genome; c1 = PvCel1; AMA11 = P. amarum (Youngstrom 11); and B2.1 indicates sequence type B2, for which we recovered 1 clone. (TIF) [file pone.0038702.s007.tif]

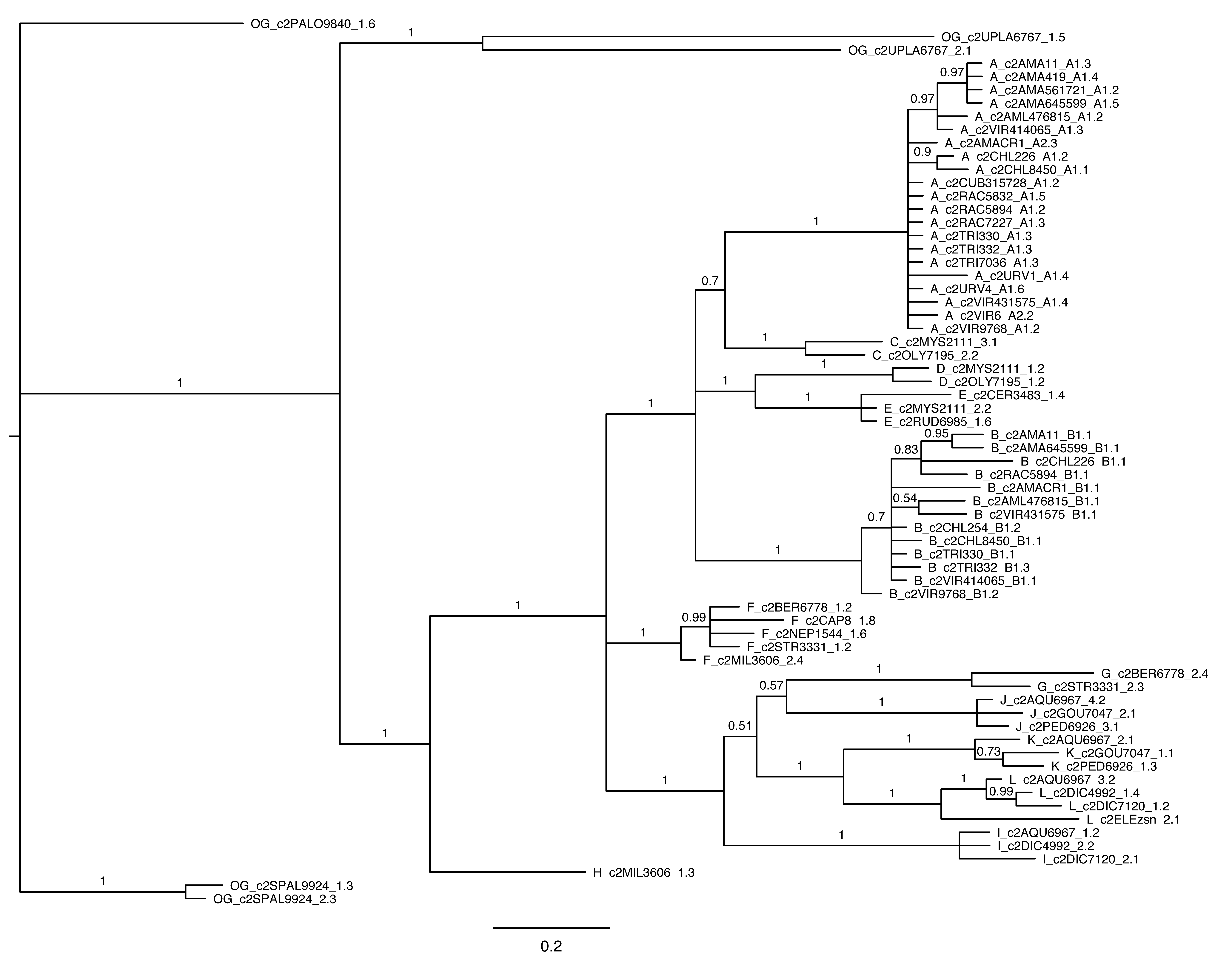

Supplement: Figure S8 — PvCel2 dataset, abridged to include fewer sequences from P. virgatum, P. amarum, and P. amarulum. Phylogram from Bayesian analyses; numbers above branches indicate posterior probabilities above 0.5. Taxon labels are in the format: B_c2CHL254_B1.2 where B_ indicates that the sequence belongs to the B genome; c2 = PvCel2; CHL254 = P. chloroleucum (Cialdella 254); and B1.2 indicates sequence type B1, for which we recovered 2 clones. (TIF) [file pone.0038702.s008.tif]
