# Supplementary material for: Five Nuclear Loci Resolve the Polyploid History of Switchgrass (Panicum virgatum L.) and Relatives
Source: PLoS One. 2012 Jun 18;7(6):e38702. doi: 10.1371/journal.pone.0038702 (PMC3377691; doi:10.1371/journal.pone.0038702)
Supplement: Appendix S1 — List of species, abbreviated name, voucher number, herbarium, and GenBank accession numbers. Specimens with PI numbers are listed in the herbarium database with Kellogg as collector. Material at UM-St. Louis is indicated by the abbreviation UMSL. (DOCX) [file pone.0038702.s009.docx]

**Appendix S1**. List of species, abbreviated name, voucher number, herbarium, and GenBank accession numbers. Specimens with PI numbers are listed in the herbarium database with Kellogg as collector. Material at UM-St. Louis is indicated by the abbreviation UMSL.

**Table S1.** Chloroplast loci

| **Species** | **Abbreviation** | **Voucher** | ***rps16-trnQ*** | ***trnC-rpoB*** |
| --- | --- | --- | --- | --- |
| *Panicum amarum* Elliott | ama7 | Youngstrom 7 (MO) | JQ947744 | JQ946894 |
| *Panicum amarum* Elliott | ama8 | Youngstrom 8 (MO) | JQ947745 | JQ946895 |
| *Panicum amarum* Elliott | ama9 | Youngstrom 9 (MO) | JQ947746 | JQ946896 |
| *Panicum amarum* Elliott | ama10 | Youngstrom 10(MO) | JQ947739 | JQ946889 |
| *Panicum amarum* Elliott | ama11 | Youngstrom 11(MO) | JQ947740 | JQ946890 |
| *Panicum amarum* Elliott | ama12 | Youngstrom 12(MO) | -- | -- |
| *Panicum amarum* Elliott | ama438 | D. Salazar 438 (UMSL) | JQ947741 | JQ946891 |
| *Panicum amarum* Elliott | ama561721 | PI 561721 (UMSL) | JQ947742 | JQ946892 |
| *Panicum amarum* Elliott | ama645599 | PI 645599 (MO) | JQ947743 | JQ946893 |
| *Panicum amarum* Elliott | amaCR1 | D. Salazar s.n. (USML) | JQ947747 | JQ946897 |
| *Panicum amarum* var. *amarulum* (Hitchc. & Chase) P. Palmer | aml419 | D. Salazar 419-1 (UMSL) | JQ947748 | JQ946898 |
| *Panicum amarum* var. *amarulum* (Hitchc. & Chase) P. Palmer | aml421901 | PI 421901 (MO) | JQ947803 | JQ946953 |
| *Panicum amarum* var. *amarulum* (Hitchc. & Chase) P. Palmer | aml476814 | PI 476814 (MO) | JQ947749 | JQ946899 |
| *Panicum amarum* var. *amarulum* (Hitchc. & Chase) P. Palmer | aml476815 | PI 476815 (MO) | JQ947750 | JQ946900 |
| *Panicum aquaticum* Poir. | aqu6967 | Zuloaga 6967 (SI) | JQ947751 | JQ946901 |
| *Panicum bergii* Arechav. | ber6778 | Zuloaga 6778 (SI) | JQ947752 | JQ946902 |
| *Panicum campestre* Nees ex Trin. | cam2129 | Filgueiras 2129 (MO) | JQ947753 | JQ946903 |
| *Panicum capillare* L. | cap8 | Triplett s.n. (JSU) | -- | -- |
| *Panicum cayennense* Lam. | cay4999 | R. Wood 4999 (RNG) | JQ947754 | JQ946904 |
| *Panicum cervicatum* Chase | cer3483 | Filgueiras 3483 (SI) | JQ947755 | JQ946905 |
| *Panicum chloroleucum* Griseb. | chl226 | Cialdella 226 (SI) | JQ947756 | JQ946906 |
| *Panicum chloroleucum* Griseb | chl254 | Cialdella 254 (SI) | JQ947757 | JQ946907 |
| *Panicum chloroleucum* Griseb | chl807 | Deginani 807 (SI) | JQ947758 | JQ946908 |
| *Panicum chloroleucum* Griseb | chl8450 | Zuloaga 8450 (SI) | JQ947759 | JQ946909 |
| *Panicum dichotomiflorum* Michx. | dic4992 | R. Wood 4992 (RNG) | JQ947761 | JQ946911 |
| *Panicum dichotomiflorum* Michx. | dic7120 | Zuloaga 7120 (SI) | JQ947762 | JQ946912 |
| *Panicum elephantipes* Nees ex Trin. | Elezsn | Zuloaga s.n. (SI) | JQ947763 | JQ946913 |
| *Panicum gouinii* E. Fourn. | gou7047 | Zuloaga 7047 (SI) | JQ947764 | JQ946914 |
| *Panicum miliaceum* L. | mil3606 | Morrone 3606 (SI) | JQ947765 | JQ946915 |
| *Panicum mystasipum* Zuloaga & Morrone | mys2111 | Filgueiras 2111 (SI) | JQ947766 | JQ946916 |
| *Panicum nephelophilum* Gaudich. | nep1544 | C. Morden 1544 (UH-Manoa) | JQ947767 | JQ946917 |
| *Panicum olyroides* Kunth | oly7195 | Zuloaga 7195 (SI) | JQ947768 | JQ946918 |
| *Panicum pedersenii* Zuloaga | ped6926 | Zuloaga 6926 (SI) | JQ947770 | JQ946920 |
| *Panicum racemosum* P. Beauv. | rac5832 | Morrone 5832 (SI) | JQ947771 | JQ946921 |
| *Panicum racemosum* P. Beauv. | rac5894 | Morrone 5894 (SI) | JQ947772 | JQ946922 |
| *Panicum racemosum* P. Beauv. | rac7227 | Zuloaga 7227 (SI) | JQ947773 | JQ946923 |
| *Panicum racemosum* P. Beauv. | raczsn1 | Zuloaga s.n. (1) (SI) | JQ947774 | JQ946924 |
| *Panicum racemosum* P. Beauv. | raczsn2 | Zuloaga s.n. (2) (SI) | JQ947775 | JQ946925 |
| *Panicum rudgei* Roem. & Schult. | rud6985 | Zuloaga 6985 (SI) | JQ947776 | JQ946926 |
| *Panicum stramineum* Hitch. & Chase | str3331 | Morrone 3331 (SI) | JQ947778 | JQ946928 |
| *Panicum tricholaenoides* Steud. | tri330 | Giussani 330 (SI) | JQ947780 | JQ946930 |
| *Panicum tricholaenoides* Steud. | tri332 | Giussani 332 (SI) | JQ947781 | JQ946931 |
| *Panicum tricholaenoides* Steud. | tri4633 | S. Renvoize 4633 (MO) | JQ947782 | JQ946932 |
| *Panicum tricholaenoides* Steud. | tri5879 | Morrone 5879 (SI) | JQ947783 | JQ946933 |
| *Panicum tricholaenoides* Steud. | tri7036 | Zuloaga 7036 (SI) | JQ947784 | JQ946934 |
| *Panicum urvilleanum* Steud. | urv1 | Youngstrom 1 (MO) | JQ947788 | JQ946938 |
| *Panicum urvilleanum* Steud. | urv4 | Youngstrom 4 (MO) | JQ947789 | JQ946939 |
| *Panicum urvilleanum* Steud. | urv309480 | PI 309480 (UMSL) | -- | -- |
| *Panicum virgatum* L. – lowland | vir6 | Youngstrom 6 (MO) | JQ947816 | JQ946966 |
| *Panicum virgatum* L. – lowland | vir441 | D. Salazar 441 (UMSL) | JQ947808 | JQ946958 |
| *Panicum virgatum* L. – lowland | vir446 | DSA-446 (UMSL) | JQ947809 | JQ946959 |
| *Panicum virgatum* L. – lowland | vir9768 | Zuloaga 9768 (SI) | JQ947820 | JQ946970 |
| *Panicum virgatum* L. – lowland | vir315723 | PI 315723 (MO) | JQ947791 | JQ946941 |
| *Panicum virgatum* L. – lowland | vir414065 | PI 414065 (MO) | JQ947795 | JQ946945 |
| *Panicum virgatum* L. – lowland | vir414070 | PI 414070 (UMSL) | JQ947799 | JQ946949 |
| *Panicum virgatum* L. – lowland | vir421521 | PI 421521 (MO) | JQ947802 | JQ946952 |
| *Panicum virgatum* L. – lowland | vir421999 | PI 421999 (UMSL) | JQ947804 | JQ946954 |
| *Panicum virgatum* L. – lowland | vir422006 | PI 422006 (MO) | JQ947805 | JQ946955 |
| *Panicum virgatum* L. – lowland | vir422016 | PI 422016 (UMSL) | JQ947806 | JQ946956 |
| *Panicum virgatum* L. – lowland | vir476291 | PI 476291 (MO) | JQ947811 | JQ946961 |
| *Panicum virgatum* L. – lowland | vir607837 | PI 607837 (UMSL) | JQ947817 | JQ946967 |
| *Panicum virgatum* L. – upland | vir16409 | Grif 16409 (UMSL) | JQ947790 | JQ946940 |
| *Panicum virgatum* L. – upland | vir315724 | PI 315724 (UMSL) | JQ947792 | JQ946942 |
| *Panicum virgatum* L. – upland | vir315725 | PI 315725 (UMSL) | JQ947793 | JQ946943 |
| *Panicum virgatum* L. – upland | vir337553 | PI 337553 (UMSL) | JQ947794 | JQ946944 |
| *Panicum virgatum* L. – upland | vir414066 | PI 414066 (UMSL) | JQ947796 | JQ946946 |
| *Panicum virgatum* L. – upland | vir414067 | PI 414067 (UMSL) | JQ947797 | JQ946947 |
| *Panicum virgatum* L. – upland | vir414069 | PI 414069 (UMSL) | JQ947798 | JQ946948 |
| *Panicum virgatum* L. – upland | vir421138 | PI 421138 (UMSL) | JQ947800 | JQ946950 |
| *Panicum virgatum* L. – upland | vir421520 | PI 421520 (MO) | JQ947801 | JQ946951 |
| *Panicum virgatum* L. – upland | vir431575 | PI 431575 (UMSL) | JQ947807 | JQ946957 |
| *Panicum virgatum* L. – upland | vir469228 | PI 469228 (MO) | JQ947810 | JQ946960 |
| *Panicum virgatum* L. – upland | vir476292 | PI476292 (UMSL) | JQ947812 | JQ946962 |
| *Panicum virgatum* L. – upland | vir476293 | PI 476293 (MO) | -- | -- |
| *Panicum virgatum* L. – upland | vir476296 | PI 476296 (UMSL) | JQ947813 | JQ946963 |
| *Panicum virgatum* L. – upland | vir476297 | PI 476297 (UMSL) | JQ947814 | JQ946964 |
| *Panicum virgatum* L. – upland | vir549094 | PI 549094 (UMSL) | JQ947815 | JQ946965 |
| *Panicum virgatum* L. – upland | vir642191 | PI 642191 (UMSL) | JQ947818 | JQ946968 |
| *Panicum virgatum* L. – upland | vir642193 | PI 642193 (UMSL) | JQ947819 | JQ946969 |
| *Panicum virgatum* var. *cubense* Griseb. | cub315728 | PI 315728 (UMSL) | JQ947760 | JQ946910 |
| *Panicum* aff. *aquaticum* | unkCR3 | D. Salazar s.n.–3 (UMSL) | JQ947786 | JQ946936 |
| *Panicum* aff. *aquaticum* | unkCR1 | D. Salazar s.n –1 (UMSL) | JQ947785 | JQ946935 |
| *Pennisetum alopecuroides* (L.) Spreng*.* | palo9840 | Park Seed 3650 (MO) | JQ947769 | JQ946919 |
| *Setaria palmifolia* (J. König.) Stapf | spal9924 | MBG 801593-2 (MO) | JQ947777 | JQ946927 |
| *Setaria viridis* (L.) P. Beauv. | svir408 | PI 408811, Doust 1405 (MO) | JQ947779 | JQ946929 |
| *Urochloa plantaginea* (Link) R. D. Webster | upla6767 | Zuloaga 6767 (SI) | JQ947787 | JQ946937 |

**Table S2.** Nuclear loci

| **Abbreviation** | ***adh*1** | ***knotted*1** | ***pabp*1** | ***PvCel*1** | ***PvCel*2** |
| --- | --- | --- | --- | --- | --- |
| palo9840 | JQ947137 | JQ947597 | JQ947334 | -- | JQ947020 |
| ama7 | JQ947070; JQ947071; JQ947072; JQ947073; JQ947074; JQ947075; JQ947076; JQ947077; JQ947078 | JQ947538; JQ947539; JQ947540; JQ947541; JQ947542 | JQ947260; JQ947261; JQ947262; JQ947263; JQ947264; JQ947265; JQ947266 | JQ947445; JQ947446; JQ947447 | JQ946977; JQ946978 |
| ama8 | -- | JQ947543; JQ947544; JQ947545; JQ947546 | -- | -- | -- |
| ama9 | -- | JQ947547; JQ947548; JQ947549 | -- | -- | -- |
| ama10 | -- | JQ947524; JQ947525; JQ947526 | -- | -- | -- |
| ama11 | JQ947055; JQ947056; JQ947057 | JQ947527; JQ947528; JQ947529 | JQ947246; JQ947247; JQ947248; JQ947249 | JQ947435; JQ947436; JQ947437 | JQ946971; JQ946972 |
| ama438 | JQ947058; JQ947059; JQ947060 | JQ947530; JQ947531 | JQ947250; JQ947251; JQ947252 | JQ947438; JQ947439 | -- |
| ama561721 | JQ947061; JQ947062; JQ947063; JQ947064; JQ947065 | JQ947532; JQ947533; JQ947534; JQ947535 | JQ947253;  JQ947254; JQ947255; JQ947256 | JQ947440; JQ947441 | JQ946973; JQ946974 |
| ama645599 | JQ947066; JQ947067; JQ947068; JQ947069 | JQ947536; JQ947537 | JQ947257; JQ947258; JQ947259 | JQ947442; JQ947443; JQ947444 | JQ946975; JQ946976 |
| amaCR1 | JQ947079; JQ947080; JQ947081; JQ947082; JQ947083 | JQ947550; JQ947551 | JQ947267; JQ947268; JQ947269 | JQ947448; JQ947449; JQ947450 | JQ946979; JQ946980; JQ946981 |
| aml419 | JQ947084; JQ947085; JQ947086 | JQ947552; JQ947553 | JQ947270; JQ947271; JQ947272; JQ947273 | JQ947451; JQ947452; JQ947453 | JQ946982; JQ946983 |
| aml421901 | JQ947204; JQ947205 | JQ947681; JQ947682; JQ947683; JQ947684 | JQ947401 | -- | -- |
| aml476814 | JQ947087 | JQ947554; JQ947555; JQ947556 | JQ947274; JQ947275; JQ947276 | -- | -- |
| aml476815 | JQ947088; JQ947089; JQ947090; JQ947091 | JQ947557; JQ947558 | JQ947277; JQ947278; JQ947279; JQ947280; JQ947281; JQ947282 | JQ947454 | JQ946984; JQ946985; JQ946986 |
| aqu6967 | JQ947092; JQ947093; JQ947094; JQ947095 | JQ947559; JQ947560; JQ947561 | JQ947283; JQ947284; JQ947285; JQ947286 | JQ947455; JQ947456 | JQ946987; JQ946988; JQ946989; JQ946990 |
| ber6778 | JQ947096; JQ947097 | JQ947562; JQ947563 | JQ947287; JQ947288 | JQ947457; JQ947458 | JQ946991; JQ946992 |
| cam2129 | JQ947098 | JQ947564 | JQ947289 | -- | -- |
| cap8 | JQ947099 | JQ947565 | JQ947290 | JQ947459 | JQ946993 |
| cay4999 | JQ947100 | JQ947566 | JQ947291 | -- | -- |
| cer3483 | JQ947101 | JQ947567 | JQ947292 | JQ947460 | JQ946994 |
| chl226 | JQ947102; JQ947103; JQ947104 | JQ947568; JQ947569; JQ947570 | JQ947293; JQ947294; JQ947295; JQ947296; JQ947297; JQ947298; JQ947299 | JQ947461; JQ947462; JQ947463 | JQ946995; JQ946996; JQ946997 |
| chl254 | JQ947105; JQ947106; JQ947107; JQ947108 | JQ947571; JQ947572 | JQ947300; JQ947301; JQ947302 | JQ947464; JQ947465; JQ947466; JQ947467 | JQ946998 |
| chl807 | JQ947109; JQ947110; JQ947111; JQ947112 | JQ947573; JQ947574; JQ947575 | JQ947303; JQ947304; JQ947305; JQ947306; JQ947307 | -- | -- |
| chl8450 | JQ947113; JQ947114; JQ947115; JQ947116; JQ947117 | JQ947576; JQ947577 | JQ947308; JQ947309; JQ947310; JQ947311; JQ947312 | JQ947468; JQ947469; JQ947470; JQ947471 | JQ946999; JQ947000; JQ947001 |
| dic4992 | JQ947122; JQ947123 | JQ947581 | JQ947317; JQ947318 | JQ947474 | JQ947004; JQ947005 |
| dic7120 | JQ947124; JQ947125 | JQ947582 | JQ947319; JQ947320; JQ947321 | JQ947475 | JQ947006; JQ947007 |
| elezsn | JQ947126; JQ947127 | JQ947583; JQ947584; JQ947585 | JQ947322; JQ947323 | JQ947476 | JQ947008; JQ947009 |
| gou7047 | JQ947128; JQ947129 | JQ947586; JQ947587; JQ947588 | JQ947324; JQ947325 | JQ947477; JQ947478 | JQ947010; JQ947011 |
| mil3606 | JQ947130; JQ947131 | JQ947589 | JQ947326; JQ947327 | JQ947479; JQ947480 | JQ947012; JQ947013 |
| mys2111 | JQ947132; JQ947133 | JQ947590; JQ947591; JQ947592 | JQ947328; JQ947329 | JQ947481; JQ947482 | JQ947014; JQ947015; JQ947016 |
| nep1544 | JQ947134 | JQ947593 | JQ947330; JQ947331 | JQ947483 | JQ947017 |
| oly7195 | JQ947135; JQ947136 | JQ947594; JQ947595; JQ947596 | JQ947332; JQ947333 | JQ947484; JQ947485 | JQ947018; JQ947019 |
| ped6926 | JQ947138 | JQ947598; JQ947599; JQ947600 | JQ947335; JQ947336; JQ947337; JQ947338 | JQ947486 | JQ947021; JQ947022; JQ947023 |
| rac5832 | JQ947139; JQ947140; JQ947141 | JQ947601; JQ947602; JQ947603 | JQ947339; JQ947340; JQ947341; JQ947342 | JQ947487; JQ947488 | JQ947024 |
| rac5894 | JQ947142; JQ947143 | JQ947604; JQ947605; JQ947606 | JQ947343; JQ947344; JQ947345 | JQ947489; JQ947490 | JQ947025; JQ947026; JQ947027 |
| rac7227 | JQ947144; JQ947145; JQ947146; JQ947147 | JQ947607; JQ947608 | JQ947346; JQ947347; JQ947348; JQ947349 | JQ947491; JQ947492 | JQ947028; JQ947029 |
| raczsn1 | -- | JQ947609; JQ947610; JQ947611 | -- | JQ947493; JQ947494; JQ947495 | -- |
| raczsn2 | -- | JQ947612; JQ947613 | JQ947350; JQ947351 | -- | -- |
| rud6985 | JQ947148 | JQ947614 | JQ947352 | JQ947496 | JQ947030 |
| str3331 | JQ947151; JQ947152 | JQ947617; JQ947618 | JQ947355; JQ947356 | JQ947499; JQ947500 | JQ947033; JQ947034 |
| tri330 | -- | JQ947620; JQ947621; JQ947622 | JQ947358 | JQ947501; JQ947502 | JQ947035; JQ947036 |
| tri332 | JQ947154; JQ947155 | JQ947623; JQ947624 | JQ947359; JQ947360 | JQ947503; JQ947504; JQ947505 | JQ947037; JQ947038 |
| tri4633 | -- | JQ947625; JQ947626 | -- | -- | -- |
| tri5879 | JQ947156; JQ947157; JQ947158 | JQ947627; JQ947628 | JQ947361; JQ947362 | JQ947506 | JQ947039 |
| tri7036 | JQ947159; JQ947160; JQ947161 | JQ947629; JQ947630; JQ947631; JQ947632 | JQ947363; JQ947364 | JQ947507; JQ947508 | JQ947040 |
| urv1 | JQ947171; JQ947172 | JQ947638; JQ947639 | JQ947373; JQ947374; JQ947375 | JQ947510; JQ947511 | JQ947043 |
| urv4 | JQ947174; JQ947175 | JQ947640; JQ947641 | JQ947376; JQ947377 | JQ947512; JQ947513 | JQ947044 |
| urv309480 | JQ947173 | -- | -- | -- | -- |
| vir6 | JQ947234; JQ947235; JQ947236 | JQ947725; JQ947726 | JQ947427; JQ947428 | JQ947518; JQ947519 | JQ947051; JQ947052 |
| vir441 | JQ947213; JQ947214; JQ947215 | JQ947696; JQ947697 | JQ947410; JQ947411 | -- | -- |
| vir446 | -- | JQ947698; JQ947699; JQ947700 | -- | -- | -- |
| vir9768 | JQ947242; JQ947243; JQ947244; JQ947245 | JQ947735; JQ947736; JQ947737; JQ947738 | JQ947431; JQ947432; JQ947433; JQ947434 | JQ947520; JQ947521; JQ947522; JQ947523 | JQ947053; JQ947054 |
| vir315723 | JQ947182; JQ947183; JQ947184 | JQ947644 | JQ947382; JQ947383; JQ947384; JQ947385 | -- | -- |
| vir414065 | JQ947187; JQ947188 | JQ947657; JQ947658 | JQ947388; JQ947389 | JQ947514; JQ947515 | JQ947045; JQ947046; JQ947047 |
| vir414070 | JQ947198; JQ947199; JQ947200 | JQ947670; JQ947671 | JQ947396; JQ947397; JQ947398 | -- | -- |
| vir421521 | JQ947201; JQ947202; JQ947203 | JQ947678; JQ947679; JQ947680 | JQ947399; JQ947400 | -- | -- |
| vir421999 | -- | JQ947685; JQ947686; JQ947687 | -- | -- | -- |
| vir422006 | JQ947206; JQ947207; JQ947208 | JQ947688; JQ947689; JQ947690 | JQ947402; JQ947403; JQ947404; JQ947405 | -- | -- |
| vir422016 | -- | JQ947691; JQ947692; JQ947693 | -- | -- | -- |
| vir476291 | JQ947221; JQ947222 | JQ947703; JQ947704; JQ947705 | JQ947414; JQ947415; JQ947416 | -- | -- |
| vir607837 | -- | JQ947727; JQ947728; JQ947729 | -- | -- | -- |
| vir16409 | JQ947176; JQ947177; JQ947178; JQ947179; JQ947180; JQ947181 | JQ947642; JQ947643 | JQ947378; JQ947379; JQ947380; JQ947381 | -- | -- |
| vir315724 | -- | JQ947645; JQ947646; JQ947647; JQ947648; JQ947649 | -- | -- | -- |
| vir315725 | JQ947185; JQ947186 | JQ947650; JQ947651; JQ947652 | JQ947386; JQ947387 | -- | -- |
| vir337553 | -- | JQ947653; JQ947654; JQ947655; JQ947656 | -- | -- | -- |
| vir414066 | JQ947189; JQ947190; JQ947191 | JQ947659; JQ947660; JQ947661 | JQ947390; JQ947391; JQ947392 | -- | -- |
| vir414067 | -- | JQ947662; JQ947663; JQ947664; JQ947665; JQ947666 | -- | -- | -- |
| vir414069 | JQ947192; JQ947193; JQ947194; JQ947195; JQ947196; JQ947197 | JQ947667; JQ947668; JQ947669 | JQ947393; JQ947394; JQ947395 | -- | -- |
| vir421138 | -- | JQ947672; JQ947673; JQ947674 | -- | -- | -- |
| vir421520 | -- | JQ947675; JQ947676; JQ947677 | -- | -- | -- |
| vir431575 | JQ947209; JQ947210; JQ947211; JQ947212 | JQ947694; JQ947695 | JQ947406; JQ947407; JQ947408; JQ947409 | JQ947516; JQ947517 | JQ947048; JQ947049; JQ947050 |
| vir469228 | JQ947216; JQ947217; JQ947218; JQ947219; JQ947220 | JQ947701; JQ947702 | JQ947412; JQ947413 | -- | -- |
| vir476292 | JQ947223; JQ947224; JQ947225; JQ947226 | JQ947706; JQ947707; JQ947708 | JQ947417; JQ947418; JQ947419; JQ947420 | -- | -- |
| vir476293 | JQ947227; JQ947228 | JQ947709; JQ947710 | JQ947421; JQ947422 | -- | -- |
| vir476296 | -- | JQ947711; JQ947712; JQ947713; JQ947714 | -- | -- | -- |
| vir476297 | -- | JQ947715; JQ947716; JQ947717; JQ947718; JQ947719 | -- | -- | -- |
| vir549094 | JQ947229; JQ947230; JQ947231; JQ947232; JQ947233 | JQ947720; JQ947721; JQ947722; JQ947723; JQ947724 | JQ947423; JQ947424; JQ947425; JQ947426 | -- | -- |
| vir642191 | JQ947237; JQ947238; JQ947239; JQ947240; JQ947241 | JQ947730; JQ947731 | JQ947429; JQ947430 | -- | -- |
| vir642193 | -- | JQ947732; JQ947733; JQ947734 | -- | -- | -- |
| cub315728 | JQ947118; JQ947119; JQ947120; JQ947121 | JQ947578; JQ947579; JQ947580 | JQ947313; JQ947314; JQ947315; JQ947316 | JQ947472; JQ947473 | JQ947002; JQ947003 |
| unkCR3 | JQ947166; JQ947167; JQ947168; JQ947169 | JQ947635; JQ947636 | JQ947368; JQ947369; JQ947370 | -- | -- |
| unkCR1 | JQ947162; JQ947163; JQ947164; JQ947165 | JQ947633; JQ947634 | JQ947365; JQ947366; JQ947367 | -- | -- |
| spal9924 | JQ947149; JQ947150 | JQ947615; JQ947616 | JQ947353; JQ947354 | JQ947497; JQ947498 | JQ947031; JQ947032 |
| svir408 | JQ947153 | JQ947619 | JQ947357 | -- | -- |
| upla6767 | JQ947170 | JQ947637 | JQ947371; JQ947372 | JQ947509 | JQ947041; JQ947042 |
